# Clinical features and prognosis of NMOSD patients with positive autoimmune antibodies

**DOI:** 10.3389/fneur.2025.1634127

**Published:** 2025-08-26

**Authors:** Yutao Liu, Yang Liu, Han Yuan, Limei Wang

**Affiliations:** Department of Neurology, The First Affiliated Hospital of Zhengzhou University, Zhengzhou University, Zhengzhou, China

**Keywords:** autoimmune antibodies, thyroid antibodies, connective tissue diseases, coexistence of autoimmune diseases, neuromyelitis optica spectrum disorders (NMOSD)

## Abstract

**Background:**

This study aimed to compare clinical outcomes in NMOSD patients with non-AQP4-IgG autoantibodies, specifically anti-connective tissue disease antibodies (anti-CTD Abs) and antithyroid antibodies (ATAbs), to evaluate their impact on disease severity and prognosis.

**Methods:**

A retrospective analysis was conducted using data from NMOSD inpatients with follow-up periods ≥180 days, stratified by antibody status: anti-CTD Abs (+)/(−), ATAbs (+)/(−), or double positivity (+)/(−). The primary outcomes included relapse rates, Expanded Disability Status Scale (EDSS) scores, and survival outcomes.

**Results:**

(1) Anti-CTD Abs (+): higher proportion of female patients, increased relapse frequency; decreased red blood cell (RBC) count and aspartate aminotransferase (AST) levels. (2) ATAbs (+): greater incidence of acute brainstem syndrome (ABS); reduced peripheral leukocyte, neutrophil, and lymphocyte counts; elevated serum urea levels. (3) Double (+): marked female predominance, higher incidence of ABS, decreased RBC count, hemoglobin (Hb) levels, and cerebrospinal fluid (CSF) chloride concentration; elevated serum urea. (4) AQP4-IgG association: AQP4-IgG-positive patients were more frequently female, with higher prevalence of anti-CTD Abs positivity but lower prevalence of ATAbs positivity. (5) Prognostic analysis: both double-positive and single-antibody-positive groups showed higher disability (EDSS ≥4.0/≥6.0) compared with antibody-negative patients, although no significant differences were observed between the two single-antibody subgroups. (6) Multivariate analysis identified combined antibody positivity (OR = 16.292), baseline EDSS score (OR = 3.179), and age at onset (OR = 1.052) as independent predictors of poor clinical outcomes.

**Conclusion:**

Routine screening for anti-CTD Abs and ATAbs in NMOSD patients may aid in assessing disease severity and prognosis. Patients with double positivity represent a high-risk subgroup requiring aggressive therapeutic strategies to prevent severe disability.

## 1 Introduction

Neuromyelitis optica spectrum disorders (NMOSDs) are rare, immune-mediated inflammatory demyelinating diseases of the central nervous system (CNS), predominantly affecting the optic nerves and spinal cord (1). Historically misclassified as a variant of multiple sclerosis, NMOSD is now recognized as a distinct nosological entity characterized by specific serological markers, particularly anti-aquaporin-4 (AQP4)-IgG antibodies (2). Accumulating evidence from neurology and rheumatology highlights frequent comorbidities with systemic autoimmune disorders, particularly connective tissue diseases (CTDs) such as systemic lupus erythematosus (SLE) and Sjögren’s syndrome (SS) (3). Population-based studies, including data from a Serbian cohort, indicate that approximately 36.8% of NMOSD patients have at least one concurrent autoimmune condition, most commonly autoimmune thyroid disease and SLE (4).

Recent advances in the understanding of NMOSD pathophysiology have further clarified its serological heterogeneity. For example, a European cohort study reported that 51.4% of patients with AQP4-IgG-positive NMOSD have serum autoantibodies associated with systemic autoimmunity (5). These include non-organ-specific autoantibodies such as antinuclear antibodies (ANAs), extractable nuclear antigens (ENAs), and anti-double-stranded DNA (anti-dsDNA) antibodies, as well as organ-specific autoantibodies such as anti-SSA/SSB, which are typically associated with CTDs (5). In addition, antithyroid antibodies, including anti-thyroglobulin (TgAb) and anti-thyroid peroxidase (TPOAb), are frequently detected in NMOSD populations (6). Although some studies suggest that the presence of these autoimmune antibodies is associated with increased disease activity, higher relapse rates, and greater disability progression (7), others have not demonstrated a statistically significant correlation (8). Therefore, the relationship between the presence of combined autoimmune antibodies and NMOSD prognosis remains inconclusive.

Although anti-CTD antibodies and antithyroid antibodies (ATAbs) are both categories of autoantibodies, they differ significantly in terms of target antigens, pathogenic mechanisms, clinical associations, and biological effects. The key distinction between anti-CTD antibodies and ATAbs lies in their organ specificity and underlying pathophysiology: the former typically cause multisystemic inflammation and tissue damage, whereas the latter primarily affect thyroid function. However, these two types of antibodies may coexist in certain conditions, such as systemic autoimmune diseases accompanied by thyroiditis, and may mutually influence the clinical course. Therefore, for research purposes, autoimmune antibodies were categorized into two groups: those associated with connective tissue diseases and those targeting the thyroid gland. This study not only examined the association between each individual antibody type and NMOSD but also investigated the impact of their coexistence on NMOSD clinical outcomes.

To address this knowledge gap, we conducted a systematic review and multidimensional comparative analysis of NMOSD cohorts stratified by serological antibody profiles: (1) anti-connective tissue disease antibodies (anti-CTD Abs), anti-CTD Abs—positive versus negative; (2) antithyroid antibodies (ATAbs)—positive versus negative; and (3) dual-positive (anti-CTD Abs + ATAbs) versus dual-negative. By analyzing demographic characteristics, clinical features, laboratory parameters, imaging findings, and antibody-related prognostic correlations across subgroups, we aimed to refine risk stratification models and inform personalized therapeutic strategies for NMOSD patients with overlapping autoimmune pathologies, thereby reducing disease burden and improving long-term outcomes.

## 2 Methods

### 2.1 Study population

We retrospectively enrolled inpatients with NMOSD admitted to the Department of Neurology at The First Affiliated Hospital of Zhengzhou University between September 2019 and June 2024 who underwent comprehensive autoimmune antibody testing. The study protocol was approved by the Institutional Review Board of our institution. Inclusion criteria were as follows: (1) diagnosis confirmed by at least two board-certified neurologists according to the 2023 update on the diagnosis and treatment of NMOSD (9). (2) Newly onset NMOSD-related clinical symptoms lasting ≥24 h were considered acute-phase cases if duration was <7 days; (3) complete testing including serum AQP4-IgG, lumbar puncture, and systemic autoimmune antibody screening, with available clinical, laboratory, and imaging data; (4) informed consent obtained from the patient or legal guardian. Exclusion criteria were as follows: (1) age <18 years; (2) Myelin oligodendrocyte glycoprotein immunoglobulin G (MOG-IgG)-positive or Glial Fibrillary Acidic Protein (GFAP)-IgG-positive status; (3) pre-existing connective tissue disease or thyroid disorder, or presence of related symptoms at enrollment; (4) disease relapse at enrollment; (5) use of systemic immunomodulators or corticosteroids within 6 months prior to enrollment; (6) concomitant neurological or visual pathway disorders that could compromise the validity of Expanded Disability Status Scale (EDSS) scoring; (7) follow-up duration <180 days.

### 2.2 Data collection

Clinical data were extracted from electronic medical records and included the following: (1) Demographic information: age, sex, and history of preceding infections; (2) Clinical characteristics: neurointensive care unit (NICU) admission, relapse history, and EDSS scores (pre-treatment and post-treatment); (3) Laboratory parameters: serum antibodies including AQP4-IgG, MOG-IgG, and GFAP-IgG concentrations were determined with a cell-based assay (CBA). Antibody titers ≥1:10 were considered as positive. Anti-CTD Abs [Anti-SSA/SSB antibodies were detected by chemiluminescent immunoassay (CLIA)]. Antibody concentrations ≥20 AU/mL were considered as positive. Antinuclear antibodies [ANAs], anti-double-stranded DNA [dsDNA] antibodies, anti-neutrophil cytoplasmic antibodies [p-ANCA/c-ANCA] and myeloperoxidase [MPO-ANCA] antibodies detected by immunofluorescence (IF). Thyroid-related parameters included thyrotropin (TSH) (Normal Range 0.56–5.91 μIU/mL), free triiodothyronine (FT3) (NR 3.28–6.47 pmol/L) and free thyroxine (FT4) (NR 7.9–18.4 pmol/L) were detected by CLIA, thyroid peroxidase antibodies (TPOAb) (NR 0–34 IU/mL) and thyroglobulin antibodies (TgAb) (NR 0–115 IU/mL) were detected by Electrochemiluminescence immunoassay (ECLIA). Cerebrospinal fluid (CSF) parameters included white blood cell (WBC) count, lymphocyte/monocyte ratio, protein concentration, glucose level, and chloride level; (4) Imaging findings: 3.0 Tesla magnetic resonance imaging (MRI) of brain and spinal cord [T2/fluid-attenuated inversion recovery (FLAIR) sequences for the brain; sagittal T2 for the spinal cord]. Imaging and clinical data were independently reviewed by two neurologists and a neuroradiologist. EDSS (range: 0–10) was used to assess disability across eight functional systems (10, 11). Relapses were defined as new or worsening neurological deficits (excluding infection-related causes) persisting >24 h or new MRI lesions occurring ≥2 months after clinical stabilization. A poor prognosis was defined as an EDSS score ≥4.0 (10, 11), and a severely poor prognosis as an EDSS score ≥6.0 (12). Follow-up data (≥180 days) were collected via telephone interviews or outpatient visits to evaluate final EDSS score, relapse status, and survival.

### 2.3 Statistical analysis

All statistical analyses were performed using SPSS v26.0 and GraphPad Prism v10.0. Continuous variables were analyzed as follows: normally distributed data were expressed as mean ± standard deviation (SD), and compared using the independent samples t-test with Levene’s test for homogeneity of variances; for non-normally distributed data, median [*M* (Q25, Q75)] was used, and comparisons were made using the Mann–Whitney U test. Categorical variables were presented as frequencies (%) and analyzed using the Chi-square test or Fisher’s exact test, as appropriate. Kaplan–Meier survival analysis was employed to evaluate prognosis, with between-group comparisons conducted using the log-rank test. Logistic regression analysis was performed to identify independent prognostic factors, starting with univariate analysis followed by multivariate modeling.

## 3 Results

### 3.1 Demographic and clinical characteristics

Among the 646 NMOSD patients initially screened, 215 (33.3%) met the inclusion criteria after application of the exclusion criteria. The mean age of the study cohort was 48.4 ± 15.6 years, with a predominance of females (165/215, 76.7%; female-to-male ratio 3.3:1). The cohort included 186 AQP4 antibody-positive patients and 29 AQP4 antibody-negative patients (positive-to-negative ratio 6.4:1). The median baseline EDSS score was 3.8 (interquartile range [IQR] 2.5–5.0), which improved to 3.0 (IQR 1.5–4.0) at the last follow-up (median follow-up duration: 497.7 ± 415.3 days). A total of 57.2% (123/215) of patients experienced disease relapses.

Clinical manifestations included the following: (1) neurological deficits: optic neuritis (ON) (59/215, 27.4%), acute myelitis (AM) (173/215, 80.5%), area postrema syndrome (APS) (23/215, 10.7%), acute brainstem syndrome (ABS) (14/215, 6.5%), diencephalic syndrome (DS) (9/215, 4.2%), and cerebral syndrome (CS) (15/215, 7.0%). (2) MRI abnormalities: brain lesions (98/215, 45.6%) and spinal cord lesions (146/215, 67.9%). (3) Treatment modalities: intravenous methylprednisolone (IVMP) monotherapy (172/215, 80.0%), IVMP combined with intravenous immunoglobulin (IVIG) (31/215, 14.4%), IVMP combined with plasma exchange (PLEX) (11/215, 5.1%), and triple therapy (1/215, 0.5%).

Autoimmune antibody profiles included the following: (1) Anti-CTD Abs were evaluated in 193 patients, of whom 84 (43.5%) tested positive. Among these, anti-SSA antibodies were the most frequently detected (59/84, 70.2%). (2) Antithyroid antibodies (ATAbs): among 172 tested patients, 61 (35.5%) were positive, with TPOAb being the most common (56/61, 91.8%). (3) Dual antibody status: among the 150 patients tested for both anti-CTD Abs and ATAbs, 21 (14.0%) were double-positive and 55 (36.7%) were double-negative. Due to the limited number of AQP4-negative patients (*n* = 29), no subgroup analysis based on AQP4 status was conducted.

### 3.2 Comparative analysis of NMOSD patients with positive versus negative anti-connective tissue disease antibodies (anti-CTD abs)

**Figure 1:**
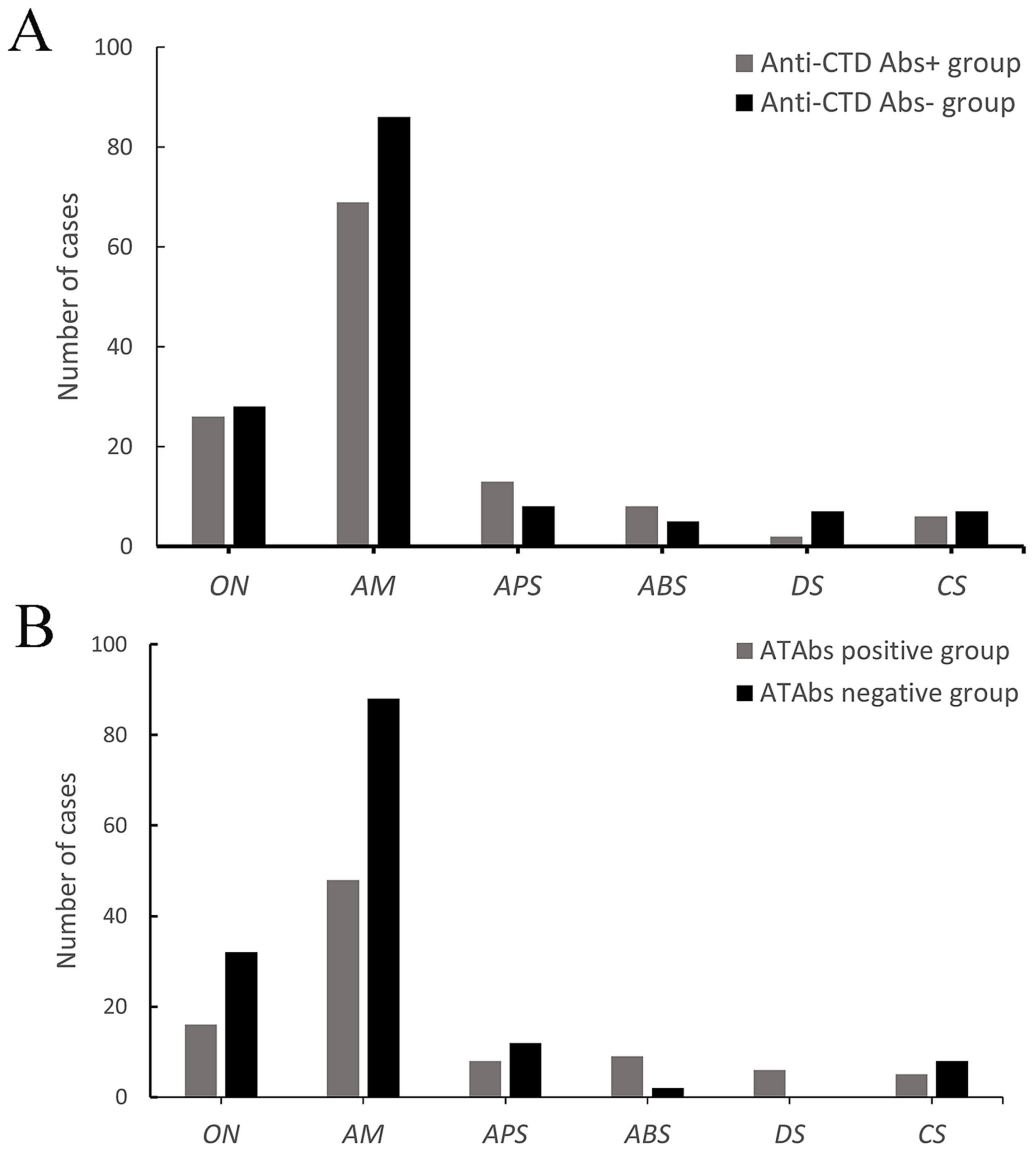
Clinical characteristics of NMOSD patients with anti-CTD Abs and ATAbs. **(A)** A comparative analysis of the clinical manifestations of NMOSD patients with positive vs. negative anti-connective tissue disease antibodies (anti-CTD Abs) was performed. Anti-CTD Ab-positive patients presented a greater prevalence of ON (26 vs. 28, 31.0% vs. 25.7%), AM (69 vs. 86, 82.1% vs. 78.9%), APS (13 vs. 8, 15.5% vs. 7.3%), ABS (8 vs. 5, 7.3% vs. 4.6%), and CS (6 vs. 7, 7.1% vs. 6.4%) but a lower incidence of DS (2 vs. 7, 2.4% vs. 6.4%). However, no significant differences in first-onset symptom distribution were found (*p* > 0.05). **(B)** A comparative analysis of the clinical manifestations of NMOSD patients with positive vs. negative antithyroid antibodies (ATAbs) was performed. ATAbs-positive patients presented a significantly greater incidence of ABS (9% vs. 2, 14.8% vs. 1.8%; *p =* 0.001). No differences were detected in ON (16 vs. 32, 26.2% vs. 28.8%), AM (48 vs. 88, 78.7% vs. 79.3%), APS (8 vs. 12, 13.1% vs. 10.8%), or CS (5 vs. 8, 8.2% vs. 7.2%) (*p* > 0.05). Anti-CTD Abs, anti-connective tissue disease antibodies; ATAbs, anti-thyroid antibodies; ON, optic neuritis; AM, acute myelitis; APS, area postrema syndrome; ABS, acute brainstem syndrome; DS, diencephalic syndrome; CS, cerebral syndrome.

Among the 193 NMOSD patients who underwent anti-CTD Abs testing, 84 (43.5%) were seropositive and 109 (56.5%) were seronegative. Demographic and clinical comparisons are summarized in Table 1 and Figure 1A.

**Table 1:** Comparison of the clinical characteristics between NMOSD patients with positive and negative anti-connective tissue disease antibodies.

Item	Total (*n* = 193)	Anti-CTD Abs positive group (*n* = 84)	Anti-CTD Abs negative group (*n* = 109)	*P*
General information				
Age (years, *x* ® ± *s*)	48.2 ± 15.1	46.9 ± 14.7	49.3 ± 15.4	0.263
Sex, female [*n* (%)]	148 (76.7)	74 (88.1)	74 (67.9)	**0.001**
Clinical symptoms, *n* (%)				
ON	54 (28.0)	26 (31.0)	28 (25.7)	0.419
AM	155 (80.3)	69 (82.1)	86 (78.9)	0.574
APS	21 (10.9)	13 (15.5)	8 (7.3)	0.072
ABS	13 (6.7)	8 (7.3)	5 (4.6)	0.703
DS	9 (4.7)	2 (2.4)	7 (6.4)	0.187
CS	13 (6.7)	6 (7.1)	7 (6.4)	0.843
Baseline EDSS	3.5 (2.5, 4.5)	3.8 (3.0, 5.0)	3.5 (2.5, 4.5)	0.179
MRI features				
Brain lesions, *n* (%)	90 (46.6)	36 (42.9)	54 (49.5)	0.356
Spinal cord lesions, *n* (%)	130 (67.4)	62 (73.8)	68 (62.4)	0.093
Blood parameters				
White blood cell count, ×10^9^/L	6.5 (4.8, 8.4)	6.7 (4.6, 9.2)	6.4 (5.0, 8.2)	0.870
Erythrocyte count, ×10^12^/L	4.2 ± 0.5	4.1 ± 0.5	4.3 ± 0.5	**0.001**
Hemoglobin, g/L	126.0 (115.0, 137.0)	122.8 (113.3, 133.8)	129.0 (118.0, 138.0)	**0.017**
Homocysteine, μmol/L	12.7 (9.5, 13.0)	12.5 (9.1, 12.9)	12.8 (9.8, 13.5)	0.330
FT4, pmol/L	13.0 (11.0, 14.4)	13.1 (11.0, 14.3)	12.9 (11.0, 14.7)	0.824
FT3, pmol/L	4.4 (3.9, 4.8)	4.4 (3.7, 4.7)	4.4 (4.1, 4.9)	0.079
TSH, μIU/mL	1.7 (0.8, 2.8)	1.7 (0.8, 2.7)	1.8 (0.9, 2.9)	0.585
ALT, U/L	17.0 (10.0, 29.6)	16.5 (10.0, 27.0)	18.0 (10.5, 31.5)	0.186
AST, U/L	17.0 (14.0, 22.6)	16.5 (14.0, 20.8)	19.0 (15.0, 24.0)	**0.009**
Urea, mmol/L	3.9 (4.9, 6.2)	5.0 (4.1, 6.6)	4.9 (3.7, 5.6)	0.191
ATAbs positivity, *n* (%)	51 (26.4)	21 (25.0)	30 (27.5)	0.925
Cerebrospinal fluid parameters				
CSF white blood cell count, ×10^6^/L	8.0 (4.0, 24.0)	10.0 (4.0, 25.5)	8.0 (2.0, 21.0)	**0.037**
CSF lymphocyte ratio, %	75.0 (68.0, 81.0)	75.0 (66.0, 80.0)	75.0 (69.0, 82.5)	0.498
CSF monocyte ratio, %	22.0 (15.0, 28.5)	22.0 (16.3, 28.0)	21.0 (15.0, 30.0)	0.787
CSF protein levels, mg/L	402.1 (291.3, 502.3)	425.8 (292.7, 541.6)	379.2 (288, 480.1)	0.222
CSF glucose levels, mmol/L	3.5 (2.9, 4.3)	3.4 (3.0, 4.2)	3.6 (3.0, 4.5)	0.211
CSF chloride levels, mmol/L	126 (123.4, 128.5)	125.8 (123.4, 128.3)	126.0 (123.4, 129.0)	0.552
Acute phase therapy, *n* (%)				
IVMP	159 (82.4)	66 (78.6)	93 (85.3)	0.222
IVMP + IVIG	23 (12.0)	12 (14.3)	11 (10.1)	0.373
IVMP + PLEX	10 (5.2)	6 (7.1)	4 (3.7)	0.336
IVMP + IVIG + PLEX	1 (0.5)	0 (0)	1 (0.9)	–
Admitted to the NICU, *n* (%)	12 (6.2)	7 (8.3)	5 (4.6)	0.285
Follow-up period (days)	509.8 ± 426.9	553.3 ± 444.3	476.3 ± 412.1	0.261
Relapse, *n* (%)	107 (55.4)	58 (69.0)	49 (45.0)	**0.001**

Anti-CTD Abs, anti-connective tissue disease antibodies; ON, optic neuritis; AM, acute myelitis; APS, area postrema syndrome; ABS, acute brainstem syndrome; DS, diencephalic syndrome; CS, cerebral syndrome; EDSS, Expanded Disability Status Scale; MRI, magnetic resonance imaging; FT4, free thyroxine; FT3, free triiodothyronine; TSH, thyrotropin; ALT, alanine aminotransferase; AST, aspartate aminotransferase; ATAbs, anti-thyroid antibodies; CSF, cerebrospinal fluid; IVMP, intravenous methylprednisolone; IVIG, intravenous immunoglobulin; PLEX, plasma exchange; NICU, neuro-intensive care unit. Bold type denotes a statistically significant difference (*p* < 0.05).

Demographics: The cohort included 148 females (76.7%), with a higher proportion of females in the anti-CTD Abs-positive group compared to the seronegative group (88.1% vs. 67.9%; *p* = 0.001). There was no significant difference in mean age at disease onset between the two groups (46.9 ± 14.7 years vs. 49.3 ± 15.4 years; *p* = 0.263).

Clinical manifestations: Anti-CTD Abs-positive patients demonstrated a higher prevalence of ON (31.0% vs. 25.7%), AM (82.1% vs. 78.9%), APS (15.5% vs. 7.3%), ABS (7.3% vs. 4.6%), and CS (7.1% vs. 6.4%), but a lower incidence of DS (2.4% vs. 6.4%). However, no statistically significant differences were observed in the distribution of initial clinical manifestations (*p* > 0.05).

Imaging features: Brain lesions were detected in 42.9% (36/84) of anti-CTD Abs-positive patients and 49.5% (54/109) of seronegative patients (*p* = 0.356). Spinal cord lesions were more frequently observed in the seropositive group (73.8% vs. 62.4%), although this difference did not reach statistical significance (*p* = 0.093).

**Figure 2:**
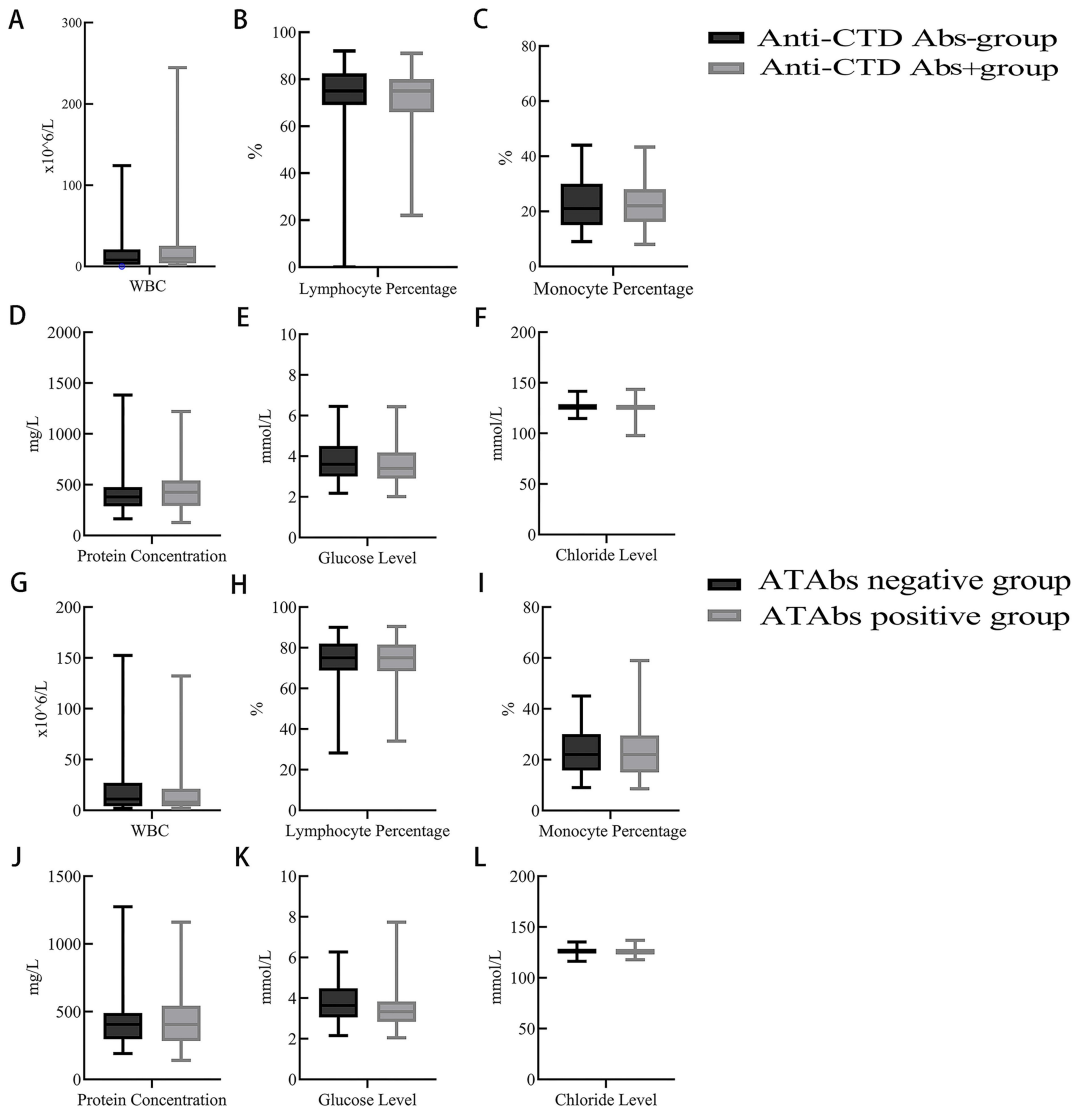
CSF parameters of NMOSD patients with anti-CTD Abs and ATAbs. **(A–F)** A comparative analysis of the CSF parameters of NMOSD patients with positive vs. negative anti-connective tissue disease antibodies (anti-CTD Abs) was performed. CSF white blood cell counts were significantly higher in the positive group compared to the negative group (*p* = 0.037) **(A)**. Other parameters, including the CSF lymphocyte ratio **(B)**, CSF monocyte ratio **(C)**, CSF protein level **(D)**, CSF glucose level **(E)**, and CSF chloride level **(F)**, were not significantly different between the two groups (*p* > 0.05). **(G–L)** A comparative analysis of the CSF parameters of NMOSD patients with positive vs. negative antithyroid antibodies (ATAbs) was performed. ATAbs-positive patients presented significantly lower CSF glucose levels [3.3 (2.8, 3.8) mmol/L vs. 3.6 (3.1, 4.5) mmol/L; *p =* 0.035] **(K)**. Other parameters (CSF white cell count) **(G)**, CSF lymphocyte ratio **(H)**, CSF monocyte ratio **(I)**, CSF protein level **(J)**, and CSF chloride level **(L)** were not significantly different between the two groups (*p* > 0.05). Anti-CTD Abs, anti-connective tissue disease antibodies; ATAbs, anti-thyroid antibodies; CSF, cerebrospinal fluid.

Laboratory parameters: Anti-CTD Abs-positive patients showed significantly lower erythrocyte counts (4.1 ± 0.5 × 10^12^/L vs. 4.3 ± 0.5 × 10^12^/L; *p* = 0.001), hemoglobin levels (122.8 [113.3, 133.8] vs. 129.0 [118.0, 138.0] g/L; *p* = 0.017), and aspartate aminotransferase (AST) activity (16.5 [14.0, 20.8] U/L vs. 19.0 [15.0, 24.0] U/L; *p* = 0.009). Cerebrospinal fluid (CSF) white blood cell counts were also elevated in seropositive patients (10.0 [4.0, 25.5] × 10^6^/L vs. 8.0 [2.0, 21.0] × 10^6^/L; *p* = 0.037). No significant differences were found between groups in CSF protein, glucose, or chloride levels, or in serum thyroid function indices (*p* > 0.05) (Table 1 and Figures 2A–F).

Treatment and outcomes: High-dose IVMP monotherapy was administered to 78.6% of seropositive patients and 85.3% of seronegative patients (*p* = 0.222). There were no significant differences in the use of IVIG or PLEX between the two groups. Relapse rates were significantly higher in seropositive patients (69.0% vs. 45.0%; *p* = 0.001). No significant differences were observed in EDSS scores or neurointensive care unit (NICU) admission rates between the groups.

Given the marked female predominance in NMOSD, the statistically significant variables previously identified were further analyzed according to gender. Among female patients, anti-CTD Abs-positive individuals had significantly lower erythrocyte counts (4.0 ± 0.4 × 10^12^/L vs. 4.2 ± 0.4 × 10^12^/L; *p* = 0.017) and AST activity (16.5 [14.0, 20.0] U/L vs. 18.0 [14.8, 24.3] U/L; *p* = 0.033). Relapse rates were also significantly higher in anti-CTD Abs-positive females (70.3% vs. 44.6%; *p* = 0.002). These findings were consistent with the overall group comparisons. However, no significant differences were observed in hemoglobin levels or CSF white blood cell counts. No statistically significant differences were found in any of the originally significant variables among anti-CTD Abs-positive male patients (Supplementary Table 1).

Notably, since the normal reference range for AST is 0–40 U/L, the observed intergroup difference in AST levels, although statistically significant, falls within the normal range and may lack clinical relevance.

### 3.3 Comparative analysis of NMOSD patients with positive vs. negative antithyroid antibodies (ATAbs)

Among 172 NMOSD patients tested for ATAbs, 61 (35.5%) were seropositive, and 111 (64.5%) were seronegative. Key comparative findings are summarized in Table 2 and Figure 1B.

**Table 2:** Comparison of the clinical characteristics between NMOSD patients with positive and negative antithyroid antibodies.

Item	Total (*n* = 172)	ATAbs positive group (*n* = 61)	ATAbs negative group (*n* = 111)	*P*
General information				
Age (years, *x* ® ± *s*)	47.8 ± 15.1	45.2 ± 14.6	49.2 ± 15.2	0.062
Sex, female [*n* (%)]	134 (77.9)	49 (80.3)	85 (76.6)	0.570
Clinical symptoms, *n* (%)				
ON	48 (27.9)	16 (26.2)	32 (28.8)	0.716
AM	136 (79.1)	48 (78.7)	88 (79.3)	0.972
APS	20 (11.6)	8 (13.1)	12 (10.8)	0.652
ABS	11 (6.4)	9 (14.8)	2 (1.8)	**0.001**
DS	6 (3.5)	6 (9.8)	0 (0)	–
CS	13 (7.6)	5 (8.2)	8 (7.2)	0.773
Baseline EDSS	3.5 (2.5, 4.5)	3.5 (2.8, 4.5)	3.5(2.5, 4.5)	0.859
MRI features				
Brain lesions, *n* (%)	81 (47.1)	27 (44.3)	54 (48.6)	0.581
Spinal cord lesions, *n* (%)	118 (68.6)	43 (70.5)	75 (67.6)	0.693
Blood parameters				
White blood cell count, ×10^9^/L	6.5 (4.9, 9.2)	5.9 (4.5, 8.0)	7.1 (5.2, 9.5)	**0.005**
Erythrocyte count, ×10^12^/L	4.2 (3.9, 4.5)	4.1 (3.8, 4.5)	4.3 (3.9, 4.6)	0.111
Hemoglobin, g/L	124.7 (115, 137.3)	123 (114.5, 131.5)	126 (116, 138)	0.155
Platelets, ×10^9^/L	241.75 ± 69.47	237.59 ± 74.88	244.04 ± 66.56	0.647
Neutrophil count, ×10^9^/L	4.3 (2.9, 6.4)	3.5 (2.7, 5.4)	4.9 (3.2, 7.2)	**0.010**
Lymphocyte count, ×10^9^/L	1.6 (1.2, 2.0)	1.4 (1.1, 1.8)	1.6 (1.3, 2.1)	**0.032**
Monocytes count, ×10^9^/L	0.4 (0.3, 0.6)	0.4 (0.3, 0.6)	0.4 (0.3, 0.6)	0.732
FT3, pmol/L	4.4 (3.9, 4.9)	4.4 (3.9, 4.9)	4.4(3.9, 4.8)	0.522
FT4, pmol/L	12.4 (10.8, 14.2)	12 (10.8, 14.0)	12.7 (10.8, 14.4)	0.173
TSH, μIU/mL	1.5 (0.7, 2.7)	1.5 (0.7, 2.8)	1.4 (0.8, 2.7)	0.960
Urea, mmol/L	4.9 (3.8, 5.9)	5.3 (4.2, 6.6)	4.7 (3.6, 5.7)	**0.007**
Creatinine, mmol/L	53.0 (45.0, 63.0)	52.1 (44.5, 64.5)	53.0 (45.0, 62.0)	0.503
ALT, u/L	17.0 (10.0, 32.9)	17.0 (10, 32.9)	17.0 (11, 32.9)	0.950
AST, u/L	17.0 (14.0, 23.8)	17.0 (14, 23.9)	18.0 (14.0, 23.0)	0.871
Connective tissue diseases antibody positive, *n* (%)	65 (37.8)	21 (34.4)	44 (39.6)	0.537
Cerebrospinal fluid parameters				
CSF white blood cell count, ×10^9^/L	10.0 (4.0, 22.8)	8.0 (4.0, 21.0)	12.0 (4.0, 26.0)	0.237
CSF lymphocyte ratio, %	75.0 (69.0, 81.8)	75.0 (68.5, 81.5)	75.0 (69.0, 82.0)	0.836
CSF monocyte ratio, %	22.0 (15.3, 29.8)	22.0 (15.0, 29.5)	22.0 (16.0, 30.0)	0.840
CSF protein levels, mg/L	404.5 (292.1, 505.3)	405.2 (284.3, 542.9)	403.7 (297.8, 487.5)	0.982
CSF glucose levels, mmol/L	3.5 (2.9, 4.3)	3.3 (2.8, 3.8)	3.6 (3.1, 4.5)	**0.035**
CSF chloride levels, mmol/L	126 (123.4, 128.5)	124.8 (123.0, 128.6)	126.2 (123.9, 128.5)	0.289
Acute phase therapy, *n* (%)				
IVMP	137 (80.0)	44 (72.1)	93 (83.8)	0.106
IVMP + IVIG	24 (14.0)	10 (16.4)	14 (12.6)	0.649
IVMP + PLEX	10 (5.8)	6 (9.8)	4 (3.6)	0.183
IVMP + IVIG + PLEX	1 (0.58)	1 (1.6)	0 (0)	–
Admitted to the NICU, *n* (%)	9 (5.2)	2 (3.3)	7 (6.3)	0.620
Follow-up period (days)	321 (186, 595)	270 (186, 533)	341 (191, 724)	0.261
Relapse, *n* (%)	98 (57.0)	32 (52.5)	66 (59.5)	0.375

ATAbs, anti-thyroid antibodies; ON, optic neuritis; AM, acute myelitis; APS, area postrema syndrome; ABS, acute brainstem syndrome; DS, diencephalic syndrome; CS, cerebral syndrome; EDSS, Expanded Disability Status Scale; MRI, magnetic resonance imaging; FT3, free triiodothyronine; FT4, free thyroxine; TSH, thyrotropin; ALT, alanine aminotransferase; AST, aspartate aminotransferase; CSF, cerebrospinal fluid; IVMP, intravenous methylprednisolone; IVIG, intravenous immunoglobulin; PLEX, plasma exchange; NICU, neuro-intensive care unit. Bold type denotes a statistically significant difference (*p* < 0.05).

Demographics: The study cohort included 134 females (77.9%). No significant differences were observed in sex distribution (80.3% vs. 76.6%; *p* > 0.05) or mean age at onset (45.2 ± 14.6 years vs. 49.2 ± 15.2 years; *p* > 0.05) between the two groups.

Clinical manifestations: ATAbs-positive patients exhibited a significantly higher incidence of ABS (14.8% vs. 1.8%; *p* = 0.001). No significant differences were found between groups in the frequency of ON (26.2% vs. 28.8%), AM (78.7% vs. 79.3%), APS (13.1% vs. 10.8%), or CS (8.2% vs. 7.2%) (*p* > 0.05 for all comparisons).

Imaging features: Brain lesions, predominantly involving the medulla, and spinal cord lesions, primarily affecting cervical or thoracic segments, were similarly distributed between the groups (brain lesions: 44.3% vs. 48.6%; spinal cord lesions: 70.5% vs. 67.6%; *p* > 0.05 for both).

Laboratory parameters: ATAbs-positive patients exhibited significantly lower white blood cell counts (5.9 [4.5, 8.0] × 10^9^/L vs. 7.1 [5.2, 9.5] × 10^9^/L; *p* = 0.005), neutrophil counts (3.5 [2.7, 5.4] × 10^9^/L vs. 4.9 [3.2, 7.2] × 10^9^/L; *p* = 0.010), lymphocyte counts (1.4 [1.1, 1.8] × 10^9^/L vs. 1.6 [1.3, 2.1] × 10^9^/L; *p* = 0.032), and CSF glucose levels (3.3 [2.8, 3.8] mmol/L vs. 3.6 [3.1, 4.5] mmol/L; *p* = 0.035). Blood urea levels were significantly higher in seropositive patients (5.3 [4.2, 6.6] mmol/L vs. 4.7 [3.6, 5.7] mmol/L; *p* = 0.007). Other parameters, including CSF cell counts, thyroid function indices, and liver enzyme levels, did not differ significantly between the groups (*p* > 0.05) (Table 2 and Figures 2G–L).

Treatment and outcomes: Treatment regimens (IVMP monotherapy: 72.1% vs. 83.8%; IVMP plus IVIG: 16.4% vs. 12.6%; IVMP plus PLEX: 9.8% vs. 3.6%) and NICU admission rates (3.3% vs. 6.3%) were comparable between the groups (*p* > 0.05). Similarly, no significant differences were observed in relapse rates (52.5% vs. 59.5%) or progression of EDSS scores.

Because of the same reason, the statistically significant items seen above were reanalyzed according to gender subgroup. It was found that ATAbs-positive female patients exhibited a significantly higher incidence of ABS (14.3% vs. 2.4%; *p* = 0.021) and blood urea levels (5.3 [4.3, 6.6] mmol/L vs. 4.6 [3.6, 5.6] mmol/L; *p* = 0.004). ATAbs-positive female patients exhibited significantly lower white blood cell counts (5.3 [4.3, 7.3] × 10^9^/L vs. 6.8 [4.9, 9.5] × 10^9^/L; *p* = 0.005), neutrophil counts (3.2 [2.5, 4.6] × 10^9^/L vs. 4.3 [3.0, 6.5] × 10^9^/L; *p* = 0.007), and lymphocyte counts (1.4 [1.1, 1.8] × 10^9^/L vs. 1.6 [1.3, 2.1] × 10^9^/L; *p* = 0.045). The results of these items were consistent with the overall two-group comparison results without gender previously. However, CSF glucose levels did not differ significantly between the groups (*p* = 0.051). None of the items that were originally statistically different showed significant differences in ATAbs-positive male patients (Supplementary Table 2).

However, the normal range of blood urea level is 2.2–8.2 mmol/L; therefore, the difference in blood urea values between the two groups, although statistically significant, falls within the normal range and may not be clinically significant.

### 3.4 Comparative analysis of NMOSD patients with double-positive vs. double-negative antibody status

Among the 150 NMOSD patients tested for both ATAbs and anti-CTD Abs, 21 (14.0%) were double-positive (positive for both antibodies), and 55 (36.7%) were double-negative (negative for both antibodies). The key comparisons are summarized in Table 3.

**Table 3:** Comparison of the clinical characteristics between the double-positive and double-negative groups of NMOSD patients with concurrently positive anti-CTD Abs and ATAbs.

Item	Total (*n* = 76)	Double-positive group (*n* = 21)	Double-negative group (*n* = 55)	*P*
General information				
Sex, female [*n* (%)]	63 (82.9)	21 (100)	42 (76.4)	**0.006**
Clinical symptoms, *n* (%)				
ON	26 (34.2)	10 (47.6)	16 (29.1)	0.128
AM	57 (75)	14 (66.7)	43 (78.2)	0.300
APS	6 (7.9)	4 (19.0)	2 (3.6)	0.050
ABS	3 (3.9)	3 (14.3)	0 (0)	–
DS	1 (1.3)	1 (4.8)	0 (0)	–
CS	4 (5.3)	1 (4.8)	0 (0)	–
Baseline EDSS	3.5 (2.5, 4.5)	3.5 (3.0, 5.0)	4.0 (2.0, 4.5)	0.278
MRI features				
Brain lesions, *n* (%)	37 (48.7)	9 (42.9)	28 (50.9)	0.530
Spinal cord lesions, *n* (%)	50 (65.8)	15 (71.4)	35 (63.6)	0.522
Blood parameters				
White blood cell count, ×10^9^/L	7.0 (5.0, 9.5)	5.9 (4.2, 8.0)	7.0 (5.0, 9.5)	0.130
Erythrocyte count, ×10^12^/L	4.2 ± 0.4	4.0 ± 0.4	4.3 ± 0.4	**0.007**
Hemoglobin, g/L	126.5 ± 14.2	119.1 ± 12.5	129.4 ± 13.9	**0.004**
Platelets, ×10^9^/L	246.5 ± 67.9	228.7 ± 69.3	253.3 ± 66.7	0.160
Neutrophil count, ×10^9^/L	4.0 (3.0, 6.3)	3.4 (2.5, 6.0)	4.0 (3.0, 6.3)	0.087
Lymphocyte count, ×10^9^/L	1.8 ± 1.6	2.0 ± 2.9	1.8 ± 0.7	0.061
Monocytes count, ×10^9^/L	0.5 ± 0.3	0.5 ± 0.5	0.4 ± 0.2	0.368
Homocysteine, μmol/L	12.3 ± 6.8	9.6 ± 2.4	13.3 ± 7.6	**0.009**
FT3, pmol/L	4.0 (4.0, 5.0)	4.3(3.5, 5.0)	4.0 (4.0, 5.0)	0.240
FT4, pmol/L	12.0 (11.0, 14.4)	11.6 (10.3, 14.0)	12.0 (11.0, 14.4)	0.161
TSH, μIU/mL	2.0 (1.0, 3.0)	1.4 (0.6, 4.0)	2.0 (1.0, 3.0)	0.938
Urea, mmol/L	4.0 (3.0, 5.3)	5.4 (4.1, 7.0)	4.0 (3.0, 5.3)	**0.014**
Cerebrospinal fluid parameters				
CSF white blood cell count, ×10^6^/L	8.0 (2.0, 20.0)	8.0 (4.0, 24.0)	8.0 (2.0, 20.0)	0.718
CSF lymphocyte ratio, %	76.0 (69.0, 83.0)	74.0 (65.5, 82.0)	76.0 (69.0, 83.0)	0.554
CSF monocyte ratio, %	19.0 (15.0, 28.5)	23.5 (14.0, 28.0)	19.0 (15.0, 28.5)	0.701
CSF protein levels, mg/L	333.0 (285.0, 458.1)	361.1 (263.0, 491.0)	333.0 (285.0, 458.1)	0.902
CSF glucose levels, mmol/L	3.7 ± 1.0	3.3 ± 0.8	3.9 ± 1.0	0.110
CSF chloride levels, mmol/L	127.0 (125.0, 129.0)	124.0 (122.8, 127.0)	127.0 (125.0, 129.0)	**0.038**
Acute phase therapy, *n* (%)				
IVMP	65 (85.5)	16 (76.2)	49 (89.1)	0.287
IVMP + IVIG	8 (10.5)	3 (14.3)	5 (9.1)	0.509
IVMP + PLEX	3 (3.9)	2 (9.5)	1 (1.8)	0.230
Follow-up period (days)	513.9 ± 419.7	572.3 ± 443.3	491.6 ± 412.4	0.384
Relapse, *n* (%)	40 (52.6)	13 (61.9)	27 (49.1)	0.317

ON, optic neuritis; AM, acute myelitis; APS, area postrema syndrome; ABS, acute brainstem syndrome; DS, diencephalic syndrome; CS, cerebral syndrome; EDSS: Expanded Disability Status Scale; MRI, magnetic resonance imaging; FT3, free triiodothyronine; FT4, free thyroxine; TSH, thyrotropin; CSF, cerebrospinal fluid; IVMP, intravenous methylprednisolone; IVIG, intravenous immunoglobulin; PLEX, plasma exchange. Bold type denotes a statistically significant difference (*p* < 0.05).

Demographics: The cohort included 63 females (82.9%), with a significantly higher proportion of females in the double-positive group compared to the double-negative group (100% vs. 76.4%; *p* = 0.006).

Clinical and laboratory parameters: Hematological indices: double-positive patients exhibited significantly lower erythrocyte counts (4.0 ± 0.4 × 10^12^/L vs. 4.3 ± 0.4 × 10^12^/L; *p* = 0.007) and hemoglobin levels (119.1 ± 12.5 g/L vs. 129.4 ± 13.9 g/L; *p* = 0.004). Metabolic markers: homocysteine (HCY) levels were significantly lower in double-positive patients (9.6 ± 2.4 μmol/L vs. 13.3 ± 7.6 μmol/L; *p* = 0.009), whereas blood urea levels were significantly higher (5.4 [4.1, 7.0] mmol/L vs. 4.0 [3.0, 5.3] mmol/L; *p* = 0.014). CSF parameters: CSF chloride levels were significantly lower in double-positive patients (124.0 [122.8, 127.0] mmol/L vs. 127.0 [125.0, 129.0] mmol/L; *p* = 0.038).

Clinical manifestations, imaging, and outcomes: No significant differences were found between groups regarding initial clinical presentations (e.g., ON: 47.6% vs. 29.1%, *p* = 0.128), prevalence of brain or spinal cord lesions (brain lesions: 42.9% vs. 50.9%; spinal cord lesions: 71.4% vs. 63.6%), relapse rates (61.9% vs. 49.1%), or treatment patterns (*p* > 0.05 for all).

Similar statistically significant parameters identified in the preceding analysis were further evaluated within gender-based subgroups. Notably, double-positive female patients demonstrated significantly reduced erythrocyte counts (4.0 ± 0.4 × 10^12^/L vs. 4.3 ± 0.4 × 10^12^/L; *p* = 0.039) and hemoglobin concentrations (119.1 ± 12.4 g/L vs. 126.2 ± 13.3 g/L; *p* = 0.045). Additionally, blood urea levels were significantly elevated in this group (5.4 [4.1, 6.9] mmol/L vs. 4.0 [3.5, 5.1] mmol/L; *p* = 0.011). Cerebrospinal fluid (CSF) chloride concentrations were also significantly lower among double-positive female patients (124.4 [122.8, 132.1] mmol/L vs. 127.2 [124.7, 129.5] mmol/L; *p* = 0.020). However, no statistically significant difference was observed in HCY levels between the groups (*p* = 0.111). Due to the absence of male individuals in the double-positive cohort, intergroup statistical comparisons based on gender could not be conducted (Supplementary Table 3).

Notably, the normal reference range for blood urea is 2.2–8.2 mmol/L; thus, although statistically significant, the observed intergroup difference in urea levels falls within the physiological range and may lack clinical relevance.

### 3.5 Distribution of autoimmune antibodies in the AQP4-IgG-seropositive population

In this study, we evaluated 150 patients diagnosed with NMOSD who underwent aquaporin-4 immunoglobulin G (AQP4-IgG) testing in conjunction with dual autoimmune antibody screening (anti-CTD Abs and ATAbs). Patients were categorized into AQP4-IgG-seropositive (*n* = 124) and AQP4-IgG-seronegative (*n* = 26) groups. Autoimmune antibody positivity was defined as the presence of at least one positive antibody (mono-anti-CTD Abs, mono-ATAbs, or dual-positive), whereas dual-negative cases were classified as antibody negative. The principal findings are summarized in Table 4: (1) demographics: the AQP4-IgG-seropositive group included a significantly higher proportion of females (82.3% vs. 57.7%; *p* = 0.006). (2) Antibody profiles: anti-CTD Abs: mono-anti-CTD Ab positivity was significantly more prevalent in AQP4-IgG-seropositive patients (33.1% vs. 11.5%; *p* = 0.028). ATAbs: mono-ATAbs positivity was significantly less frequent in the seropositive cohort (16.1% vs. 38.5%; *p* = 0.010). The prevalence of dual antibody positivity did not differ significantly between the two groups (14.5% vs. 11.5%; *p* > 0.05). (3) Clinical correlates: no significant differences were observed in age distribution or rates of antibody negativity between the groups (*p* > 0.05).

**Table 4:** Comparison of the autoimmune antibody distributions between the AQP4-IgG- seropositive and negative NMOSD patient groups.

Item	Total (*n* = 150)	AQP4-IgG seropositive group (*n* = 124)	AQP4-IgG seronegative group (*n* = 26)	*P*
Age (years, *x* ® ± *s*)	47.0 ± 14.4	47.3 ± 14.3	48.7 ± 15.1	0.811
Sex, female [n (%)]	117 (78.0)	102 (82.3)	15 (57.7)	**0.006**
Autoimmune antibody-positive, *n* (%)				
Mono-anti-CTD Abs-positive	44 (29.3)	41 (33.1)	3 (11.5)	**0.028**
Mono-ATAbs-positive	30 (20.0)	20 (16.1)	10 (38.5)	**0.010**
Dual antibody-positive	21 (14.0)	18 (14.5)	3 (11.5)	0.685
Autoimmune antibody-negative, *n* (%)				
Dual antibody-negative	55 (36.7)	45 (36.3)	10 (38.5)	0.835

Anti-CTD Abs, anti-connective tissue disease antibodies; ATAbs, antithyroid antibodies. Bold type denotes a statistically significant difference (*p* < 0.05).

### 3.6 Prognostic analysis of NMOSD patients: role of autoimmune antibodies

A total of 150 NMOSD patients were classified according to autoimmune antibody status (autoimmune antibody-positive: mono-anti-CTD Abs, mono-ATAbs, or dual-positive; autoimmune antibody-negative: dual-negative) to assess prognostic implications. Poor clinical outcome was defined as an EDSS score ≥4.0 at the final follow-up, with severely poor prognosis defined as EDSS ≥6.0. Baseline characteristics and clinical outcomes are summarized in Table 5.

**Table 5:** Comparison of the baseline characteristics between the good-prognosis and poor-prognosis groups of NMOSD patients.

Item	Total (*n* = 150)	Good prognosis group (*n* = 109)	Poor prognosis group (*n* = 41)	*P*
Sex, *n* (%)				
Female	117 (78.0)	84 (77.1)	33 (80.5)	0.625
Male	33 (22.0)	25 (22.9)	8 (19.5)	
Age (years, *x* ® ± *s*)	47.5 ± 14.4	45.5 ± 13.6	52.8 ± 15.2	**0.006**
Clinical symptoms, *n* (%)				
ON	43 (28.7)	35 (32.1)	8 (19.5)	0.128
AM	118 (78.7)	82 (75.2)	36 (87.8)	0.094
APS	18 (12.0)	13 (11.9)	5 (12.2)	>0.999
ABS	10 (6.7)	8 (7.3)	2 (4.9)	0.728
DS	6 (4.0)	1 (0.9)	5 (12.2)	**0.002**
CS	11 (7.3)	7 (6.4)	4 (9.8)	0.493
Baseline EDSS	3.5 (2.5, 4.5)	3.0 (3.0, 4.0)	5.0 (4.0, 6.5)	**<0.00**1
Follow-up period (days)	329.5 (184.3, 692.3)	341.0 (192.0, 763.5)	229.0 (188.5, 537.5)	0.246
Last follow-up EDSS	2.5 (2.0, 4.0)	2.0 (2.0, 3.0)	4.5 (4.0, 6.0)	**<0.001**
MRI features				
Brain lesions	73 (48.7)	53 (48.6)	20 (48.8)	0.986
Spinal cord lesions	102 (68.0)	71 (65.1)	31 (75.6)	0.220
Autoimmune antibody, *n* (%)	95 (63.3)	59 (54.1)	36 (87.8)	**<0.001**
Acute phase therapy, *n* (%)				
IVMP	124 (82.7)	94 (86.2)	30 (73.2)	0.060
IVMP + IVIG	16 (10.7)	10 (9.2)	6 (14.6)	0.377
IVMP + PLEX	9 (6.0)	5 (4.6)	4 (9.8)	0.257
IVMP + PLEX + IVIG	1 (0.7)	0 (0)	1 (2.4)	0.273

ON, optic neuritis; AM, acute myelitis; APS, area postrema syndrome; ABS, acute brainstem syndrome; DS, diencephalic syndrome; CS, cerebral syndrome; EDSS, Expanded Disability Status Scale; MRI, magnetic resonance imaging; IVMP, intravenous methylprednisolone; IVIG, intravenous immunoglobulin; PLEX, plasma exchange. Bold type denotes a statistically significant difference (*p* < 0.05).

Baseline characteristics: no significant differences were observed between prognosis groups with respect to gender distribution, treatment modalities (IVMP/IVIG/PLEX combinations), spinal cord involvement, or cerebral lesion burden (*p* > 0.05). However, patients with poor prognosis exhibited a significantly higher mean age at disease onset (52.8 ± 15.2 vs. 45.5 ± 13.6 years; *p* = 0.006). Autoimmune antibody positivity was significantly more prevalent in the poor prognosis group (87.8% vs. 54.1%; *p* < 0.001). EDSS scores: both baseline and follow-up EDSS scores were markedly higher in the poor prognosis cohort (*p* < 0.001).

Due to significant gender differences in NMOSD, the statistically significant variables identified previously were further analyzed according to gender subgroups. In female patients, the poor prognosis group had a significantly older mean age at onset compared to the good prognosis group (54.8 ± 14.7 vs. 45.4 ± 14.0 years; *p* = 0.002). The positivity rate of autoimmune antibodies was significantly higher in the poor prognosis group (87.9% vs. 54.8%; *p* = 0.001). Both baseline and follow-up EDSS scores were significantly elevated in the poor prognosis group (*p* < 0.001). In the male subgroup, the poor prognosis group also exhibited higher baseline (*p* = 0.042) and follow-up EDSS scores (*p* < 0.001). However, no statistically significant differences were observed for other originally significant variables in the male poor prognosis group (Supplementary Table 4).

**Figure 3:**
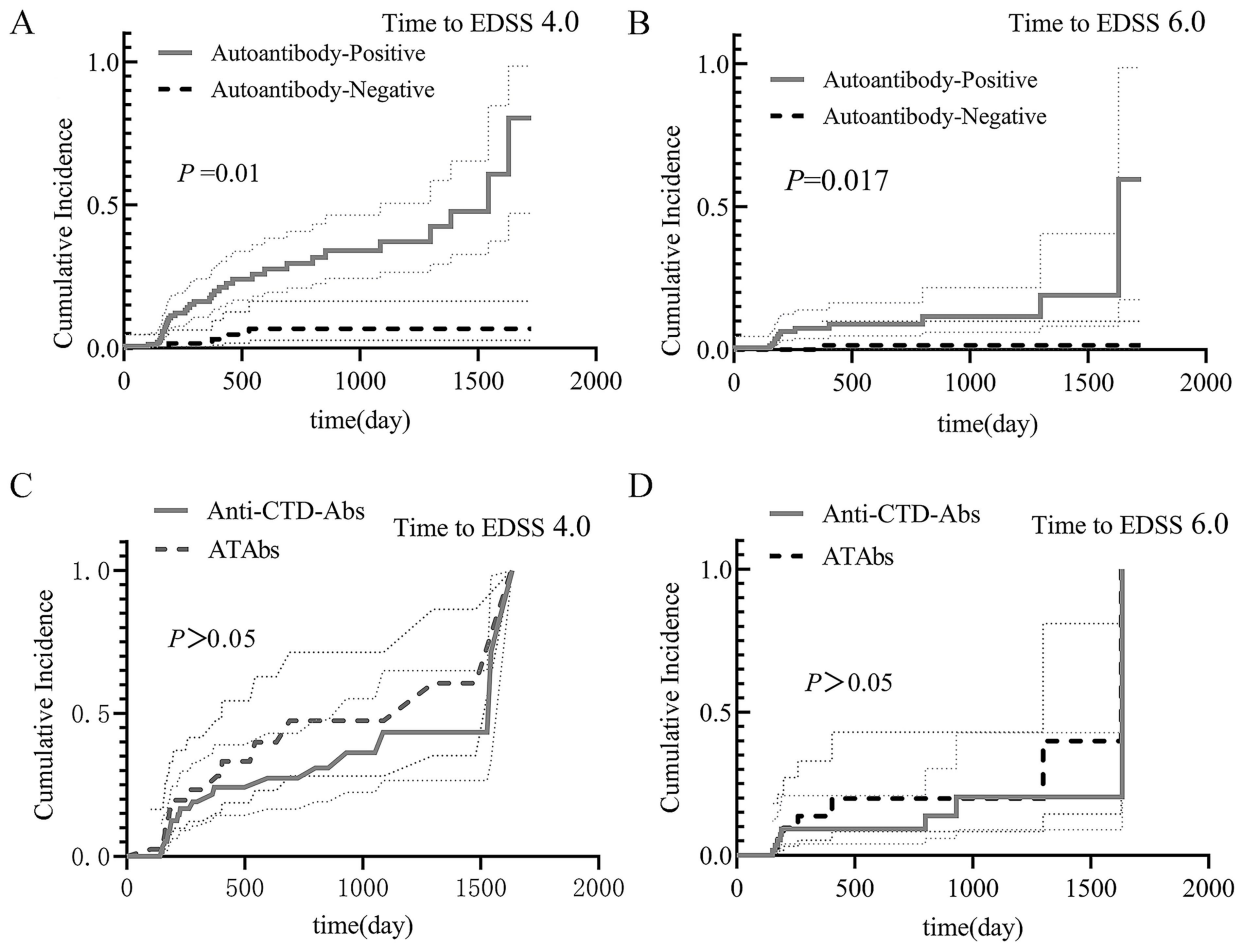
Prognostic analysis of NMOSD patients. The results of the Kaplan–Meier analysis are shown. **(A)** Poor prognosis (EDSS score ≥4.0): the antibody-positive group had a significantly greater cumulative incidence (log-rank *p =* 0.01). **(B)** Severely poor prognosis (EDSS score ≥6.0): the antibody-positive group also had an elevated risk (log-rank *p =* 0.017). **(C,D)** Mono-antibody subgroups: no significant differences were found in outcomes between the mono-anti-CTD Ab and mono-ATAb groups (*p* > 0.05). Anti-CTD Abs, anti-connective tissue disease antibodies; ATAbs, anti-thyroid antibodies; EDSS, Expanded Disability Status Scale.

Prognostic outcomes: The median follow-up duration was 329.5 days (IQR 184.3–692.3), with no significant difference between the antibody-positive and antibody-negative cohorts (*p* = 0.246). Kaplan–Meier analysis demonstrated: (1) For poor prognosis (EDSS score ≥4.0), the antibody-positive group showed a significantly higher cumulative incidence (log-rank *p* = 0.01; Figure 3A). (2) For severely poor prognosis (EDSS score ≥6.0), the antibody-positive group also had a higher risk (log-rank *p* = 0.017; Figure 3B). (3) In the mono-antibody subgroups, no significant differences were observed between the mono-anti-CTD Ab and mono-ATAb groups (*p* > 0.05; Figures 3C,D).

Prognostic factor analysis: Univariate logistic regression identified four significant predictors of poor prognosis (Table 6): (1) Presence of autoimmune antibodies (OR = 6.102, 95% CI: 2.226–16.727); (2) Age at onset (OR = 1.038, 95% CI: 1.010–1.066); (3) Baseline EDSS score (OR = 3.122, 95% CI: 2.127–4.583); and (4) CSF protein levels (OR = 1.003, 95% CI: 1.001–1.004). Multivariate logistic regression confirmed that the presence of autoimmune antibodies was the strongest independent risk factor (OR = 16.292, 95% CI: 3.525–75.291; *p* < 0.001), followed by baseline EDSS score (OR = 3.179) and age at onset (OR = 1.052) (Table 7).

**Table 6:** Univariate logistic regression analysis of the good-prognosis group vs. the poor-prognosis group.

Prognostic factors	β	OR	Lower limit	Upper limit	*P*
Autoimmune antibody-positive	1.809	6.102	2.226	16.727	**<0.001**
Female	0.205	0.815	0.334	1.988	0.652
Age	0.037	1.038	1.010	1.066	**0.007**
ON	0.668	0.513	0.215	1.224	0.132
TM	0.863	2.371	0.845	6.651	0.101
Baseline EDSS	1.139	3.122	2.127	4.583	**<0.001**
Brain lesions	0.006	1.006	0.491	2.064	0.986
Spinal cord lesions	0.506	1.659	0.735	3.746	0.223
CSF protein levels	0.003	1.003	1.001	1.004	**0.002**

ON, optic neuritis; TM, transverse myelitis; EDSS, Expanded Disability Status Scale; CSF, cerebrospinal fluid. Bold type denotes a statistically significant difference (*p* < 0.05).

**Table 7:** Multivariate logistic regression analysis of the good-prognosis group vs. the poor-prognosis group.

Prognostic Factors	*β*	OR	Lower limit	Upper limit	*P*
Autoimmune antibody-positive	2.791	16.292	3.525	75.291	**<0.001**
Baseline EDSS	1.156	3.179	2.041	4.950	**<0.001**
Age	0.050	1.052	1.009	1.096	**0.016**
CSF protein levels	0.001	1.001	0.999	1.003	0.330

EDSS, Expanded Disability Status Scale; CSF, cerebrospinal fluid. Bold type denotes a statistically significant difference (*p* < 0.05).

## 4 Discussion

Neuromyelitis optica spectrum disorder (NMOSD) is a rare, relapsing neuroinflammatory astrocytopathy characterized by a marked female predominance. Epidemiological studies have reported a female-to-male ratio of approximately 4:1, with a peak incidence occurring between 40 and 59 years of age (13). Among the 215 eligible patients in this study, 165 (76.7%) were female, corresponding to a female-to-male ratio of 3.3:1. The mean age at symptom onset was 48.4 ± 15.6 years, which is slightly higher than previously reported values. This discrepancy may be attributable to differences in sample size, geographic distribution, or referral bias across studies.

Acute transverse myelitis, presenting as lower limb or tetraplegic paralysis, was the most common clinical manifestation, observed in 80.5% of patients, followed by ON in 27.4% of cases. These findings are consistent with global epidemiological data, which indicate that NMOSD predominantly affects the spinal cord and optic nerves (14). Similarly, diencephalic involvement was infrequent in our cohort (4.2%), a finding comparable to a previous study of a Belgian cohort that reported diencephalic involvement in 3.4% of cases, primarily manifesting as narcolepsy (15).

Evidence suggests that NMOSD typically follows a relapsing–remitting course, with relapses occurring at intervals ranging from months to years (16). Most relapses clinically present as either ON or transverse myelitis (TM), with ON more commonly observed in patients under 30 years of age and TM more prevalent in those over 30 years (17). Patients who are AQP4-IgG seropositive face a 60% risk of clinical relapse within 1 year following an episode of myelitis, highlighting the prognostic significance of serostatus (18). In this study, 57.2% (123/215) of patients experienced relapses during follow-up, a rate consistent with the high recurrence rate characteristic of NMOSD.

Aquaporin-4 immunoglobulin G (AQP4-IgG), a T-cell-dependent IgG1 autoantibody derived from peripheral plasma cells, is part of a polyclonal autoantibody repertoire (19). Its pathogenic mechanism involves binding to AQP4 expressed in peripheral tissues such as skeletal muscle, kidneys, and lungs, thereby initiating inflammatory responses via complement activation and neutrophil extracellular trap formation (20). This systemic autoimmune response may explain the increased co-occurrence of other autoimmune disorders, including Sjögren’s syndrome (SS) and autoimmune thyroid disease (AITD), among patients with NMOSD (4). The presence of such comorbidities enhances diagnostic certainty for NMOSD, as neurological manifestations are more likely attributable to NMOSD rather than vasculitic complications associated with SLE or SS (9). Mendelian randomization analyses have demonstrated causal associations between AITD, SLE, SS, and susceptibility to NMOSD, although these autoimmune conditions do not appear to influence each other’s onset (21).

Moreover, NMOSD exhibits a distinct autoimmune profile frequently accompanied by CTD-related comorbidities. In a cohort of 37 patients with AQP4-IgG-seropositive NMOSD, 51.4% tested positive for additional pathogenic autoantibodies, with ANA being the most prevalent, followed by anti-dsDNA antibodies (5). Xie et al. (22) conducted a retrospective analysis of inpatients over a 5-year period; however, the follow-up duration was not clearly defined. Their findings indicated that SS was the most common CTD in NMOSD-CTD overlap cases (10/16, 62.5%), with significantly elevated AQP4-IgG positivity in serum (100%) and CSF (70.2%; *p* = 0.009). Additionally, higher rates of serum ANA, anti-SSA, and anti-Ro52 autoantibodies were observed in NMOSD-CTD patients (*p* < 0.001) (22). Similarly, Zhang et al. (23) performed a retrospective analysis involving inpatients over a 10-year period, with a median follow-up of 3.4 years (IQR: 1.8–7.2) and a maximum follow-up of 20.3 years. They reported higher titers of anti-SSA and anti-AQP4 antibodies in NMOSD patients compared to healthy controls (*p* < 0.05), as well as a significantly higher relapse rate among patients with CTD-NMOSD overlap (75.0% vs. 25.0%; *p* < 0.01) (23).

Conversely, Lee et al. (8), in another retrospective study involving patients hospitalized over a 10-year period with a median follow-up of 6.7 years (IQR: 3.6–9.5), evaluated the clinical and prognostic significance of ANAs in patients with NMO-IgG-positive NMOSD. Multidimensional assessment of relapse rate, MRI lesion burden, and prognosis in relation to ANA status revealed that although ANAs were associated with certain autoimmune features (e.g., presence of other autoantibodies and systemic disease manifestations), they did not significantly influence disease activity or long-term prognosis in NMOSD. These findings suggest that ANAs should not be considered a primary biomarker for predicting disease progression (8).

Given these conflicting conclusions, we re-evaluated the impact of anti-CTD Abs on disease severity and prognosis in NMOSD patients. Importantly, our study uniquely included both ATAbs and anti-CTD Abs, analyzing the effects of isolated positivity for either antibody type, dual positivity, or dual negativity on disease outcomes. In our cohort, anti-SSA antibodies were the most frequently detected anti-CTD Abs (70.2%), while ATAbs were predominantly represented by TPOAb (91.8%). Other autoantibodies identified included anti-SSB, anti-cyclic citrullinated peptide (anti-CCP), and anti-dsDNA antibodies. The high prevalence of anti-SSA/TPOAb coexistence suggests shared immunopathogenic mechanisms between NMOSD and CTDs. We found that the proportions of patients with EDSS scores ≥4.0 and ≥6.0 were higher in both the dual-positive and single-positive groups compared with the dual-negative group, indicating an association between antibody presence and greater disease severity. Furthermore, regression analysis demonstrated that antibody positivity was linked to poorer prognosis.

The discrepancies observed across the aforementioned studies may be attributed to several factors: (1) differences in study populations—these studies were conducted in different countries and may reflect ethnic variations, which could be further explored through larger multicenter, multinational collaborations in future research; and (2) differences in observation periods. Our study focused on the acute phase, with a follow-up duration of 6 months, whereas Xie et al. (22) did not specify the follow-up period, Zhang et al. (23) reported a median follow-up of 3.4 years, and Lee et al. (8) described a median follow-up of 6.7 years. It is plausible that these autoantibodies exert a more pronounced effect during the early stages of the disease, with their long-term impact lacking statistical significance.

Numerous studies in the fields of rheumatology and neurology have documented the coexistence of NMOSD with CTDs. CTDs, such as SLE, SS, rheumatoid arthritis (RA), and interstitial lung disease (ILD), are characterized by multi-organ autoimmunity and distinct autoantibody profiles (e.g., ANA, anti-Jo-1, and anti-Ro/La) (24). In our study, 43.52% of 193 NMOSD patients tested positive for anti-CTD Abs, with a higher proportion of females in the anti-CTD-positive group compared to the anti-CTD-negative group (88.1% vs. 67.9%; *p* = 0.001). This observed sex disparity may be explained by the following factors: (1) sex-specific immune regulation: females demonstrate enhanced adaptive immune responses, including increased B-cell activity and antibody production, which are modulated by estrogen-mediated pathways (25, 26); (2) X chromosome genetics: the female X chromosome harbors multiple immune-related genes (e.g., TLR7 and FOXP3), which may increase susceptibility to autoimmune disorders (27); and (3) hormonal modulation: estrogen enhances cytokine production (e.g., IL-6 and TNF-α) and autoantibody titers following vaccination, potentially contributing to heightened autoimmune reactivity (28).

A retrospective analysis of 525 NMOSD patients demonstrated that individuals with positive ANA results were more likely to exhibit higher EDSS scores during both the acute phase and final follow-up. Additionally, patients with positive ANA had a greater proportion of severe acute myelitis episodes, combined episodes of severe acute myelitis and optic neuritis, and increased motor and visual disabilities compared to those with negative ANA. These findings suggest that ANA positivity may be associated with greater disease severity and disability in NMOSD, indicating potential utility as a prognostic biomarker (7). Similarly, another retrospective study found that positive ANA was associated with increased disease activity in NMOSD patients. Patients with positive ANA exhibited a shorter disease duration and a significantly reduced time from onset to an EDSS score of 4.0, further supporting the hypothesis that ANA positivity may serve as a marker of poor prognosis (29). Our study also observed similar trends regarding relapse rates, with significantly higher relapse rates in NMOSD patients who tested positive for anti-CTD Abs compared to those who tested negative (69.0% vs. 45.0%; *p* = 0.001). Relapse rates remained significantly higher when reanalyzed according to gender subgroups among seropositive female patients (70.3% vs. 44.6%; *p* = 0.002). However, in contrast to the aforementioned studies, no significant between-group differences were observed in EDSS scores. This discrepancy may be attributable to the relatively small sample size of 193 NMOSD patients who underwent anti-CTD Abs testing, underscoring the need for larger studies to further investigate the relationship between antibody status and disease severity, including EDSS scores.

Potential mechanisms underlying autoantibody-mediated pathogenesis include the following: (1) blood–brain barrier (BBB) disruption: Anti-CTD Abs may bind to endothelial antigens, thereby increasing BBB permeability (30). This facilitates the entry of AQP4-IgG into CNS, where it binds to astrocyte foot processes, initiating inflammatory cascades, such as complement activation and neutrophil extracellular trap formation, ultimately resulting in astrocyte injury and demyelination (31). (2) Neurodegenerative effects: Anti-SSA/Ro antibodies, a subset of anti-CTD Abs, can bind to surface SSA antigens on neural cells, leading to axonal degeneration and myelin loss. This impairs neural conduction and exacerbates neurological disability, particularly in brainstem and spinal cord lesions (32). (3) Inflammatory modulation: Anti-CTD Abs promote the upregulation of proinflammatory cytokines, such as IL-6 and TNF-*α*, thereby intensifying intrathecal inflammation. This may act synergistically with AQP4-IgG-induced astrocyte damage, accelerating relapse frequency and disability progression (33). These potential mechanisms remain under investigation, with no definitive conclusions established to date, and further evidence is required for validation.

Thyroglobulin (Tg) and thyroid peroxidase (TPO) are key autoantigens in AITDs, generated through CD4 + helper T cell-mediated B cell responses (33). The association between NMOSD and AITDs may involve shared pathogenic mechanisms mediated by CD4 + T cells (34). A retrospective analysis of 108 children with NMOSD further demonstrated that the proportion of females, EDSS scores, longitudinally extensive transverse myelitis (LETM) rate, ANA positivity, and MOG antibody positivity were significantly higher in the ATAbs-positive group compared to the ATAbs-negative group. The elevated serum TPOAb and TgAb positivity rates in NMOSD patients relative to healthy controls support a link between ATAbs levels and NMOSD pathogenesis. These findings are both statistically significant and clinically relevant, suggesting that ATAbs positivity may serve as a marker of more severe neurological deficits and poorer prognosis in pediatric NMOSD (6). These findings differ from those of our study, in which 61 (35.5%) of 172 NMOSD patients were serum ATAbs positive, with no significant differences observed in relapse rate (52.5% vs. 59.5%) or EDSS progression patterns. The lack of differences in relapse rate and EDSS scores may be associated with the relatively low ATAbs positivity rate in our cohort. Although this low positivity rate may be attributable to our limited sample size, we plan to expand the cohort to further investigate the relationship between ATAbs status and NMOSD clinical characteristics.

However, the mechanisms by which ATAbs contribute to NMOSD pathogenesis remain incompletely understood. Several hypotheses have been proposed: (1) molecular mimicry: Tg contains seven antigenic regions, with the first antigenic site (amino acids 84–149) harboring a VDAEG motif that may cross-react with the VDAQG motif (amino acids 143–168) of myelin basic protein (MBP). This molecular mimicry may drive the production of autoantibodies against MBP, thereby promoting demyelination (35). (2) Immune complex formation: ATAbs may form immune complexes with MBP, indirectly compromising myelin integrity. For instance, anti-MBP antibodies induced by ATAbs can exacerbate demyelination, as demonstrated in experimental autoimmune encephalomyelitis (EAE) models (35). (3) Immune cell crosstalk: ATAbs are secreted by autoreactive B cells activated by CD4 + T helper (Th) cells. In NMOSD, CD4 + T cell cultures have been correlated with EDSS scores, suggesting that Th cell-driven autoimmunity may amplify the disease process (34). (4) Thyroid hormone dysregulation: Thyroid hormones (THs) are essential for oligodendrocyte differentiation and CNS remyelination. ATAbs may interfere with TH synthesis (e.g., via thyroiditis), thereby impairing remyelination and accelerating disability progression (36). (5) AQP4 antibody cross-reactivity: AQP4, a key biomarker in NMOSD, is expressed in thyroid follicular cells. AQP4-IgG may cross-react with thyroid antigens, leading to thyroid dysfunction and amplifying systemic autoimmunity (37). Of course, these potential mechanisms are still under investigation and not yet conclusive, requiring further evidence to support them.

Demographic comparisons: No significant differences were observed in gender distribution between the ATAbs -positive and ATAbs -negative groups (female predominance: 80.3% vs. 76.6%), consistent with the overall female predominance in the cohort (female-to-male ratio, 3.3:1). The median age at onset was comparable between the two groups (45.2 ± 14.6 years vs. 49.2 ± 15.2 years; *p* = 0.062). These findings align with domestic studies reporting that NMOSD typically manifests between the fourth and fifth decades of life (38).

Clinical manifestations: Spinal cord involvement (e.g., myelitis) was the most common clinical presentation in both groups (78.7% vs. 79.3%). However, ATAbs-positive patients exhibited a higher proportion of ABS as the initial presentation compared to ATAbs-negative patients (14.8% vs. 1.8%; *p* = 0.001). Furthermore, ATAbs-positive female patients demonstrated a significantly higher incidence of ABS compared with ATAbs-negative female patients (14.3% vs. 2.4%; *p* = 0.021) upon subgroup analysis by gender. This observation contrasts with previous reports suggesting predominant spinal cord involvement in AITD-associated NMOSD (21), potentially due to differences in sample size or regional diagnostic practices. No significant differences were observed in brain lesion frequency (44.3% vs. 48.6%) or spinal cord segment involvement (70.5% vs. 67.6%) between the two groups (*p* > 0.05), further supporting the phenotypic overlap between ATAbs-positive and ATAbs-negative NMOSD patients (21).

Mechanistic considerations: The absence of sex differences in ATAbs seropositivity may reflect the dual role of estrogen in immune regulation; although estrogen enhances B-cell activation and autoantibody production (21), its immunomodulatory effects may balance disease susceptibility across sexes. The comparable age at onset further suggests that ATAbs positivity does not significantly alter disease initiation but may influence the severity of clinical presentation.

Females were more frequently represented in the double-positive cohort compared to the double-negative cohort, which may be attributed to the potent immunomodulatory effects of estrogen (39, 40). However, no significant differences were observed between groups in the incidence of MRI-confirmed cerebral lesions or relapse. All participants received standardized induction therapy with high-dose methylprednisolone pulse regimen. As a second-generation synthetic glucocorticoid, methylprednisolone exerts immunosuppressive effects via lipophilic transmembrane penetration, forming cytoplasmic receptor-ligand complexes that inhibit the NF-κB signaling pathway (41). This glucocorticoid has become the standard treatment for acute NMOSD relapse.

Mechanistic studies revealed a dual mechanism of action: (1) transcriptional suppression of proinflammatory mediators, including TNF-*α*, IL-6, and GM-CSF, through GR-mediated chromatin remodeling, thereby reducing demyelination in the spinal cord and optic nerve white matter tracts (42); and (2) modulation of the complement pathway by inhibiting C3 convertase activity and membrane attack complex formation, which helps maintain the integrity of the blood–brain barrier (BBB) (43).

Serum hematological parameters, including red blood cell counts (4.1 ± 0.5 × 10^12^/L vs. 4.3 ± 0.5 × 10^12^/L, *p* = 0.001), hemoglobin levels (122.8 [113.3, 133.8] g/L vs. 129.0 [118.0, 138.0] g/L, *p* = 0.017), and AST activity (16.5 [14.0, 20.8] U/L vs. 19.0 [15.0, 24.0] U/L, *p* = 0.009), showed significant differences between NMOSD patients with anti-CTD Abs positivity and seronegative controls. However, the clinical relevance of these hematological abnormalities in the pathophysiology of antibody-mediated NMOSD requires further comprehensive biomolecular investigation (3).

Hematological analyses revealed distinct sex-related patterns: the lower hemoglobin levels observed in anti-CTD Abs-positive patients may reflect the higher proportion of females in this cohort (88.1% vs. 67.9%, *p* = 0.001), given the well-documented sex dimorphism in erythropoiesis (3). Cerebrospinal fluid (CSF) cytological analysis following lumbar puncture demonstrated significant inflammatory differences, with anti-CTD Abs-positive patients showing higher CSF white blood cell counts (10.0 [4.0, 25.5] × 10^6^/L vs. 8.0 [2.0, 21.0] × 10^6^/L, *p* = 0.037), consistent with findings from domestic cohort studies indicating intrathecal immune activation in antibody-positive NMOSD patients (3). This CSF pleocytosis correlates with disease severity (23). However, no significant differences were found in hemoglobin levels or CSF white blood cell counts when reanalyzed according to gender subgroups, suggesting inconsistency with the overall intergroup comparison without gender stratification. Therefore, the clinical significance of these findings requires further clarification through studies with larger sample sizes.

Pathophysiological correlations extended to CSF biochemical parameters: (1) elevated CSF protein levels (425.8 [292.7, 541.6] mg/L vs. 379.2 [288, 480.1] mg/L, *p* = 0.222) likely reflect a combination of mechanisms involving BBB disruption, as indicated by increased albumin quotient; and (2) inflammatory mediators derived from activated T cells and macrophages may contribute to protease-induced dysfunction of the blood-CSF barrier (1, 34). These findings support emerging evidence suggesting that CSF protein elevation in NMOSD is associated with an increased risk of accumulating permanent disability (1).

Distinct biochemical profiles were observed between NMOSD patient subgroups. Compared with seronegative controls, ATAbs-positive patients exhibited significantly lower CSF glucose concentrations. CSF glucose homeostasis primarily relies on BBB transport mechanisms, which remain stable under physiological conditions (44). Evidence suggests that thyroid dysfunction may impair CSF glucose metabolism by altering astrocytic lactate shuttle activity and monocarboxylate transporter regulation (44). The ATAbs-positive subgroup demonstrated marginally reduced peripheral blood cell counts, including white blood cells (5.9 [4.5, 8.0] vs. 7.1 [5.2, 9.5] × 10^9^/L; *p* = 0.005), neutrophils (3.5 [2.7, 5.4] vs. 4.9 [3.2, 7.2] × 10^9^/L; *p* = 0.010), and lymphocytes (1.4 [1.1, 1.8] vs. 1.6 [1.3, 2.1] × 10^9^/L; *p* = 0.032). ATAbs-positive female patients also exhibited significantly lower white blood cell counts (5.3 [4.3, 7.3] × 10^9^/L vs. 6.8 [4.9, 9.5] × 10^9^/L; *p* = 0.005), neutrophil counts (3.2 [2.5, 4.6] × 10^9^/L vs. 4.3 [3.0, 6.5] × 10^9^/L; *p* = 0.007), and lymphocyte counts (1.4 [1.1, 1.8] × 10^9^/L vs. 1.6 [1.3, 2.1] × 10^9^/L; *p* = 0.045). These findings may indicate thyroid hormone modulation of hematopoietic stem cell differentiation pathways (45, 46). These observations correspond with clinical evidence demonstrating that thyroid dysfunction, which occurs in 23–38% of NMOSD patients, correlates with disease severity and therapeutic response (45).

Compared with the double-negative group, the double-positive subgroup (ATAbs +/anti-CTD Abs+) exhibited distinct hematological features, including reduced hemoglobin (119.1 ± 12.5 vs. 129.4 ± 13.9 g/L; *p* = 0.004) and red blood cell counts (4.0 ± 0.4 vs. 4.3 ± 0.4 × 10^12^/L; *p* = 0.007). Double-positive patients also exhibited significantly lower erythrocyte counts (4.0 ± 0.4 × 10^12^/L vs. 4.3 ± 0.4 × 10^12^/L; *p* = 0.039) and hemoglobin levels (119.1 ± 12.4 g/L vs. 126.2 ± 13.3 g/L; *p* = 0.045). These differences may arise from two synergistic mechanisms: (1) chronic inflammation-induced hepcidin upregulation impairs iron mobilization; (2) sex-specific hematological variations (100% female prevalence in the double-positive group vs. 76.4% in the negative group).

The NMOSD with negative AQP4 antibodies may be caused by multiple mechanisms and may involve various confounding factors, thereby presenting distinct clinical features and treatment responses. AQP4-negative NMOSD shows significant differences from AQP4-positive NMOSD in terms of clinical manifestations, imaging features, treatment response and prognosis. The following are its main differences:

Clinical manifestations and antibody subtype: (1) AQP4-positive NMOSD is highly specific, with approximately 80% of NMOSD patients being AQP4 antibody positive. Common core symptoms include optic neuritis, long-segment transverse myelitis (LETM), APS (intractable nausea and vomiting), diencephalic syndrome (hyponatremia, drowsiness). Women are predominantly affected (female-to-male ratio 9–11: 1) (2). (2) AQP4-negative NMOSD: MOG-AD: More common in children and young adults, with a relatively balanced female-to-male ratio (1–2: 1). Optic neuritis often involves the anterior segment of the optic nerve, accompanied by optic disk edema (90%), and is prone to bilateral simultaneous involvement. Myelitis can present as long or short segments, with more frequent involvement of the lumbar spinal cord and conus medullaris. Some patients may present with acute disseminated encephalomyelitis (ADEM)-like symptoms (47). GFAP antibody-related diseases: May present as myelitis (in approximately 50% of cases), with more frequent involvement of the thoracic spinal cord and less frequent involvement of the lumbar spinal cord. Some patients may develop optic neuritis, although imaging may not reveal obvious abnormalities (48). Other antibodies (e.g., NMDAR, GlyR, GAD): Rare and may present as atypical encephalitis or myelitis.

Imaging features: (1) AQP4-positive NMOSD: Spinal cord lesions are predominantly longitudinally extensive (≥3 vertebral segments), with prominent involvement of the central gray matter. Intracranial lesions typically occur in regions with high AQP4 expression, including the area postrema, diencephalon, and periventricular regions (2). (2) AQP4-negative NMOSD: MOG-AD: MRI in optic neuritis demonstrates enhancement of the anterior optic nerve, frequently accompanied by optic nerve sheath inflammation. Spinal cord lesions may exhibit a “sagittal linear hypersignal” or “pseudo-ependymal dilation sign.” Intracranial involvement commonly affects the thalamus, basal ganglia, and pia mater (47). GFAP astrocytic disease: Spinal cord lesions predominantly involve the thoracic region, with pericentral canal enhancement (48). Other rare antibody-associated NMOSD: For instance, NMDAR encephalitis with associated myelitis may present with T2 hyperintensity in the ventral gray matter of the spinal cord.

Therapeutic response and prognosis: (1) AQP4-positive NMOSD: Demonstrates favorable response to B-cell targeted therapies (e.g., inebilizumab, anti-CD19 monoclonal antibody), with a 77% risk reduction. Immunosuppressive agents [e.g., mycophenolate mofetil (MMF)] have been shown to reduce relapse rates (1). (2) AQP4-negative NMOSD: MOG-AD: Exhibits sensitivity to corticosteroid pulse therapy, although some patients experience relapses. Prognosis is generally favorable, with mild disability; some patients achieve full recovery after a single episode (49). GFAP astrocytic disease: Responds well to corticosteroid therapy, though certain patients require long-term immunosuppression (50). Other antibody-associated NMOSD: In cases positive for NMDAR or GlyR antibodies, immunomodulatory therapy (e.g., IVIG, rituximab) may be indicated.

Recurrence rate and long-term disability: (1) AQP4-positive NMOSD: Characterized by a high relapse rate, frequently leading to permanent visual impairment or paralysis (approximately 50%). (2) AQP4-negative NMOSD: MOG-AD exhibits a low relapse rate with favorable recovery. The recurrence pattern in GFAP or other rare antibody-associated NMOSD remains incompletely defined, with potential for slow progression in some patients.

Association with autoimmune diseases: (1) AQP4-positive NMOSD: Frequently coexists with other autoimmune conditions, including Sjögren syndrome and systemic lupus erythematosus. (2) AQP4-negative NMOSD: MOG-AD is less commonly associated with systemic autoimmune disorders. GFAP astrocytic disease may occur in the context of paraneoplastic syndromes.

AQP4-negative NMOSD represents a heterogeneous group of disorders with variable clinical features, imaging characteristics, and treatment responses depending on the specific antibody subtype (e.g., MOG, GFAP, and NMDAR). Compared with AQP4-positive NMOSD, MOG-AD generally demonstrates a more favorable prognosis, whereas rare antibody-associated NMOSD may necessitate individualized therapeutic approaches.

Clinical recommendations: (1) Repeat AQP4 antibody testing, particularly during acute episodes (serum or CSF). (2) Test for MOG antibodies (approximately 20–40% of AQP4-negative NMOSD cases are MOG antibody-positive). 3. Exclude alternative diagnoses through enhanced MRI, CSF analysis (including oligoclonal bands and cell count), and systemic screening for tumors or infections. 4. Implement individualized treatment strategies: MOG-associated disease may demonstrate limited response to rituximab, necessitating tailored selection of immunosuppressive agents.

Homocysteine (HCY), a sulfur-containing non-proteinogenic amino acid, is primarily generated through the methionine metabolic pathway (51, 52). Its biosynthesis involves key enzymatic reactions catalyzed by methionine synthase and cystathionine β-synthase (53). Under physiological conditions, HCY undergoes catabolism via two major pathways: remethylation, which requires folate and vitamin B12 and transsulfuration which is dependent on vitamin B6 (54). Dysregulation of HCY metabolism resulting in hyperhomocysteinemia is strongly associated with various pathological conditions, including cardiovascular diseases and neurodegenerative disorders (55). In addition to its role in cellular methylation reactions, HCY exerts pleiotropic effects on redox homeostasis, apoptotic pathways, and cell proliferation through multiple mechanisms (56). For example, compared with healthy controls, patients with SLE exhibit significantly elevated HCY levels (57), while thyroid hormone dysregulation further modulates HCY metabolism (58). Specifically, hypothyroidism impairs HCY catabolism by reducing the enzymatic activity of methylenetetrahydrofolate reductase (MTHFR), a key enzyme in the remethylation pathway, thereby contributing to hyperhomocysteinemia (58, 59). In this study, significant differences in HCY levels were observed between the double-positive and double-negative antibody groups. However, no significant differences were found between groups regarding HCY levels (*p* = 0.111) when reanalyzed according to gender subgroups. The inconsistent results observed in the double-positive group may be attributable to the smaller sample size within this subgroup. These preliminary findings require further validation in larger cohorts to determine their clinical relevance.

A systematic review and meta-analysis demonstrated a linear association between AQP4-IgG seropositivity and female sex (60). This observation is consistent with retrospective data from 92 NMOSD patients, which showed an increased female-to-male ratio among AQP4-IgG-positive individuals (61), potentially mediated by estrogen-induced enhancement of AQP4-IgG production via activation of CD40 signaling in B cells (61). Our findings corroborate these results, revealing significantly higher monoclonal anti-CTD antibody positivity in the AQP4-IgG-positive cohort compared to the seronegative controls.

The association between AQP4-IgG and anti-CTD Abs may involve multiple immunopathogenic mechanisms: (1) molecular mimicry between AQP4 epitopes and microbial antigens triggers cross-reactive humoral responses (62); (2) co-activation of the innate immune system leads to synergistic B-cell stimulation (63); (3) shared genetic susceptibility loci (e.g., HLA-DRB1 * 03:01 variants) predispose individuals to the production of dual autoantibodies (63–65). Conversely, increased monoclonal ATAbs positivity in AQP4-IgG-negative patients may reflect distinct immunopathogenic pathways, potentially involving differential T-helper cell subset polarization (e.g., Th17/Treg imbalance), distinct cytokine profiles (e.g., IL-17 versus IFN-*γ* dominance), or epigenetic modifications influencing autoantibody repertoires. It should be noted that these mechanisms are hypothetical and require further experimental and clinical validation.

The observed link between AQP4-IgG-negative status and thyroid autoimmunity suggests that overlapping autoimmune risk alleles (e.g., CTLA-4 polymorphisms) or environmental triggers (e.g., iodine exposure) may influence self-reactivity. However, the relatively limited sample size in our study necessitates validation through large-scale multicenter cohorts and functional investigations, including: (1) refining NMOSD subphenotyping using autoantibody signatures; (2) elucidating pathogenic hierarchies between AQP4-IgG and ATAbs; (3) informing precision medicine strategies for antibody-defined disease subsets.

The EDSS remains a cornerstone for evaluating functional impairment in patients with NMOSD (10, 66). Key thresholds include the following: EDSS 4.0, characterized by significant limitation in ambulation or involvement of two functional systems (e.g., pyramidal, brainstem) (10); EDSS ≥4.0, associated with an increased risk of axonal injury and progression to irreversible disability (10); and EDSS 6.0, indicating loss of independent ambulation requiring unilateral assistive devices (67). In this study, patient prognosis was dichotomized as follows: moderate disability, defined as an EDSS score ≥4.0; and severe disability, defined as an EDSS score ≥6.0.

Our findings align with emerging evidence indicating that NMOSD patients with autoimmune antibody positivity (anti-CTD-Abs/ATAbs) demonstrate a dose-dependent increase in disability. Specifically: (1) age-related disability: Late-onset NMOSD patients (>50 years) exhibit accelerated EDSS progression, with final scores 2.3 points higher than those of early-onset patients (*p* < 0.01) (68–70). (2) Autoantibody signatures: ATAbs positivity predicts severe clinical phenotypes in pediatric NMOSD patients (OR = 3.2 for EDSS ≥6.0) (30); AQP4-IgG-positive patients with CTDs have a 1.8-fold higher relapse rate and 2.1-fold greater disability accumulation compared to their CTD-negative counterparts (29, 71). (3) Autoimmune biomarkers: Anti-SSA antibodies are independently associated with higher EDSS scores (*β* = 1.4, *p* = 0.03) (64).

Multivariate analysis identified three key prognostic determinants: age (OR = 1.052, 95% CI: 1.009–1.096, *p* = 0.016), baseline EDSS score (OR = 3.179, 95% CI: 2.041–4.950, *p* < 0.001), and autoantibody profile (OR = 16.292, 95% CI: 3.525–75.291, *p* < 0.001). These findings corroborate prior reports associating autoimmune seropositivity with accelerated disability trajectories (64, 71), while extending this framework to include age-related disability progression. The interaction between chronological aging and immune-mediated neurodegeneration warrants further investigation into potential shared pathogenic mechanisms, such as mitochondrial dysfunction and microglial activation.

This study had three principal methodological limitations: (1) design limitations: The single-center retrospective design, based on electronic medical record data extraction, may introduce selection bias and ascertainment bias due to incomplete documentation of non-investigated parameters. (2) Sample size limitations: Stringent inclusion criteria (mandatory cerebrospinal fluid analysis, inpatient thyroid antibody/functional assessments, and anti-CTD antibody testing), combined with the rarity of dual seropositive cases (concurrent anti-CTD-Abs+/ATAbs+), limited enrollment to 21 eligible patients. Among the 215 patients included, only 29 were AQP4-negative, limiting statistical power for subgroup analyses of rare antibody combinations. (3) Temporal dimension limitations: The cross-sectional design precludes longitudinal assessment of antibody titer dynamics or treatment responses, potentially obscuring causal relationships between autoantibody profiles and clinical outcomes.

To address these limitations, we propose the following future research directions: (1) multicenter prospective registries prioritizing the enrollment of dual-seropositive cohorts and comprehensive phenotyping (e.g., immune cell subsets, cytokine profiles, and MRI biomarkers); (2) longitudinal antibody kinetic studies with serial CSF/blood sampling to elucidate the timelines of autoantibody-mediated pathogenesis; (3) subgroup-specific mechanistic investigations comparing humoral-driven phenotypes (ATAbs+/anti-CTD-Abs+) versus AQP4-IgG-dominated disease; early-onset versus late-onset seropositive subgroups; (4) Integrative multiomics approaches combining epigenetic, transcriptomic, and B-cell receptor sequencing to elucidate clinical phenotype heterogeneity. These steps are critical for: (1) validating antibody profiling as a prognostic tool for NMOSD stratification, (2) identifying biomarker-guided therapeutic targets for seropositive subtypes, and (3) clarifying pathogenic overlaps between autoimmune neuroinflammation and systemic CTDs.

In clinical practice, routine autoimmune and thyroid antibody testing in patients with NMOSD, regardless of AQP4 status or the presence of a history of symptoms of autoimmune or thyroid disease, holds significant clinical value in evaluating disease severity, prognosis, and guiding subsequent pharmacological management. For patients exhibiting dual positivity for autoimmune and thyroid antibodies, which may indicate an unfavorable prognosis, more aggressive therapeutic strategies are recommended to reduce the risk of progression to more severe disease stages.

## 5 Conclusion

This study characterizes distinct autoimmune antibody profiles in NMOSD patients, demonstrating strong correlations between serological markers and disease phenotypes: (1) antibody distribution: Anti-CTD Abs are predominantly represented by anti-SSA positivity, whereas ATAbs are primarily associated with TPOAb reactivity. Dual seropositivity is observed in 14% of cases and is associated with unique demographic and clinical characteristics. (2) Sex-specific patterns: Anti-CTD Abs-positive cohorts show a marked female predominance, potentially reflecting hormonal influences on systemic autoimmunity. ATAbs-positive patients exhibit a male-predominant ABS phenotype, suggesting distinct immunopathogenic mechanisms. Double-seropositive patients demonstrate the highest female predominance, indicating that hormonal factors may influence the co-expression of autoantibodies. (3) Clinical-antibody correlations: AQP4-IgG-positive patients display distinct serological profiles. Co-existing positivity for anti-CTD Abs and ATAbs serves as a reliable prognostic biomarker. (4) Prognostic stratification: Patients in the combined-antibody-positive group exhibit worse clinical outcomes.

These findings enhance our understanding of NMOSD heterogeneity by defining antibody seropositivity thresholds for phenotypic classification, identifying sex-specific immune dysregulation patterns, and confirming the prognostic value of combined autoantibody profiles. Further studies are warranted to evaluate whether dual-seropositive patients may derive differential therapeutic benefit from B-cell depletion therapy or immunomodulatory interventions targeting overlapping autoimmune pathways.

This study represents the first systematic stratification of NMOSD patients based on ATAbs and anti-CTD Abs status, investigating the clinical features and prognosis of those with isolated anti-CTD Abs positivity, isolated ATAbs positivity, and dual positivity. It concludes that the dual-positive group exhibits more severe clinical manifestations and poorer prognosis. Routine testing for anti-CTD Abs and ATAbs in NMOSD patients contributes to accurate assessment of disease severity and prognosis. Dual positivity identifies a high-risk subgroup necessitating intensified treatment to prevent severe neurological disability.

## Data Availability

The original contributions presented in the study are included in the article/Supplementary material, further inquiries can be directed to the corresponding author.
