# Supplementary material for: Clinical features and prognosis of NMOSD patients with positive autoimmune antibodies
Source: Front Neurol. 2025 Aug 26;16:1634127. doi: 10.3389/fneur.2025.1634127 (PMC12417128; doi:10.3389/fneur.2025.1634127)
Supplement: Supplementary file 1 [file Table_1.docx]

**Supplementary Table 1.** Comparison of the clinical characteristics between NMOSD patients with positive and negative anti-connective tissue disease antibodies according to gender subgroup

| **Item** | **Total** | **Anti-CTD Abs positive group** | **Anti-CTD Abs negative group** | ***P*** |
| --- | --- | --- | --- | --- |
| Sex, female [n] | 148 | 74 | 74 |  |
| Erythrocyte count, ×10^12^/L | 4.1±0.4 | 4.0±0.4 | 4.2±0.4 | **0.017** |
| Hemoglobin, g/L | 122.0(113.0, 130.0) | 120.5 (112.0, 128.7) | 122.0 (114.0, 144.5) | 0.197 |
| AST, U/L | 17.0(14.0, 23.0) | 16.5 (14.0, 20.0) | 18.0 (14.8, 24.3) | **0.033** |
| CSF white blood cell count, ×10^6^/L | 8.0 (4.0, 20.0) | 8.0 (4.0, 24.5) | 8.0(2.0, 16.0) | 0.057 |
| Relapse, n (%) | 85 (57.4%) | 52(70.3%) | 33(44.6%) | **0.002** |
| Sex, male [n] | 45 | 10 | 35 |  |
| Erythrocyte count, ×10^12^/L | 4.6±0.4 | 4.6±0.3 | 4.5±0.4 | 0.613 |
| Hemoglobin, g/L | 139.0 (136.0, 150.5) | 143.0(138.0, 151.8) | 138.0 (133.0, 150.0) | 0.101 |
| AST, U/L | 18.0 (15.0, 22.0) | 16.5 (13.0, 19.5) | 18.0 (15.0, 23.0) | 0.218 |
| CSF white blood cell count, ×10^6^/L | 8.0 (2.0, 21.0) | 14.0(3.5, 67.0) | 8.0 (2.0, 20.0) | 0.324 |
| Relapse, n (%) | 22 (48.9%) | 6 (60.0%) | 16 (45.7%) | 0.425 |

Note​​:

​​Anti-CTD Abs: anti-connective tissue disease antibodies; ​​AST​​: Aspartate aminotransferase; CSF​​: Cerebrospinal fluid.​​
