# Supplementary material for: Clinical features and prognosis of NMOSD patients with positive autoimmune antibodies
Source: Front Neurol. 2025 Aug 26;16:1634127. doi: 10.3389/fneur.2025.1634127 (PMC12417128; doi:10.3389/fneur.2025.1634127)
Supplement: Supplementary file 2 [file Table_2.docx]

Supplementary Table 2. Comparison of the clinical characteristics between NMOSD patients with positive and negative antithyroid antibodies according to gender subgroup

| **Item** | **Total** | **ATAbs positive group** | **ATAbs negative group** | ***P*** |
| --- | --- | --- | --- | --- |
| Sex, female [n] | 134 | 49 | 85 |  |
| ABS, n(%) | 9 (6.7%) | 7 (14.3%) | 2(2.4%) | **0.021** |
| White blood cell count, ×10^9^/L | 6.0 (4.7, 8.2) | 5.3(4.3, 7.3) | 6.8(4.9, 9.5) | **0.005** |
| Neutrophil count, ×10^9^/L | 3.8(2.8, 6.0) | 3.2(2.5, 4.6) | 4.3 (3.0, 6.5) | **0.007** |
| Lymphocyte count, ×10^9^/L | 1.6(1.2, 1.9) | 1.4(1.1, 1.8) | 1.6 (1.3, 2.1) | **0.045** |
| Urea, mmol/L | 4.8(3.8, 5.8) | 5.3 (4.3, 6.6) | 4.6 (3.6, 5.6) | **0.004** |
| CSF glucose levels, mmol/L | 3.4 (2.9, 4.3) | 3.3 (2.7, 3.7) | 3.5(3.0, 4.5) | 0.051 |
| Sex, male [n] | 38 | 12 | 26 |  |
| ABS, n(%) | 2 (5.3%) | 2 (16.7%) | 0 (0.0%) | 0.094 |
| White blood cell count, ×10^9^/L | 7.5(6.4, 10.1) | 7.2 (6.1, 11.1) | 7.8(6.5, 10.1) | 0.638 |
| Neutrophil count, ×10^9^/L | 5.5(3.9, 8.0) | 5.4 (4.0, 8.1) | 5.7 (3.9, 8.0) | 0.937 |
| Lymphocyte count, ×10^9^/L | 1.6(1.3, 2.3) | 1.5 (1.0, 2.0) | 1.6 (1.3, 2.4) | 0.470 |
| Urea, mmol/L | 5.0(3.6, 6.3) | 5.1 (4.0, 6.4) | 5.0(3.5, 6.3) | 0.706 |
| CSF glucose levels, mmol/L | 3.8(3.3, 4.4) | 3.6 (2.9, 4.7) | 3.9 (3.4, 4.4) | 0.683 |

Note:

ATAbs: anti-thyroid antibodies; ABS: Acute brainstem syndrome; CSF: Cerebrospinal fluid.
