# Supplementary material for: Clinical features and prognosis of NMOSD patients with positive autoimmune antibodies
Source: Front Neurol. 2025 Aug 26;16:1634127. doi: 10.3389/fneur.2025.1634127 (PMC12417128; doi:10.3389/fneur.2025.1634127)
Supplement: Supplementary file 3 [file Table_3.docx]

Supplementary Table 3. Comparison of the clinical characteristics between the double-positive and double-negative groups of NMOSD patients with concurrently positive anti-CTD Abs and ATAbs according to gender subgroup

| **Item** | **Total** | **Double-Positive group** | **Double-Negative Group** | ***P*** |
| --- | --- | --- | --- | --- |
| Sex, female [n] | 63 | 21 | 42 |  |
| Erythrocyte count, ×10^12^/L | 4.2±0.4 | 4.0±0.4 | 4.3±0.4 | **0.039** |
| Hemoglobin, g/L | 123.8±13.3 | 119.1±12.4 | 126.2±13.3 | **0.045** |
| Homocysteine​​, μmol/L | 11.2±3.8 | 10.1±2.4 | 11.7±4.3 | 0.111 |
| Urea, mmol/L | 4.7 (3.6, 5.6) | 5.4 (4.1, 6.9) | 4.3 (3.5, 5.1) | **0.011** |
| CSF chloride levels, mmol/L | 126.3 (123.7, 129.0) | 124.4 (122.8, 132.1) | 127.2 (124.7, 129.5) | **0.020** |
| Sex, male [n] | 13 | 0 | 13 |  |
| Erythrocyte count, ×10^12^/L | 4.5±0.4 | - | 4.5±0.4 | **-** |
| Hemoglobin, g/L | 139.7±10.9 | - | 139.7±10.9 | **-** |
| Homocysteine​​, μmol/L | 17.7±11.5 | - | 17.7±11.5 | **-** |
| Urea, mmol/L | 5.2(3.2, 6.0) | - | 5.2(3.2, 6.0) | **-** |
| CSF chloride levels, mmol/L | 126.0 (123.1, 127.2) | - | 126.0 (123.1, 127.2) | **-** |

Note: CSF: Cerebrospinal fluid.
