# Supplementary material for: Clinical features and prognosis of NMOSD patients with positive autoimmune antibodies
Source: Front Neurol. 2025 Aug 26;16:1634127. doi: 10.3389/fneur.2025.1634127 (PMC12417128; doi:10.3389/fneur.2025.1634127)
Supplement: Supplementary file 4 [file Table_4.docx]

**Supplementary Table 4.** Comparison of the baseline characteristics between the good-prognosis and poor-prognosis groups of NMOSD patients according to gender subgroup

| **Item** | **Total** | **Good prognosis group** | **Poor prognosis group** | ***P*** |
| --- | --- | --- | --- | --- |
| Sex, Female (n) | 117 | 84 | 33 |  |
| Age (years, x ̅±s) | 48.0±14.8 | 45.4±14.0 | 54.8±14.7 | **0.002** |
| DS(%) | 3(2.6%) | 0 (0.0%) | 3 (9.1%) | **0.021** |
| Baseline EDSS | 3.5 (2.5, 4.5) | 3.0(2.0, 4.0) | 6.0 (4.5, 6.5) | **<0.001** |
| Last follow-up EDSS | 2.5 (2.0, 4.0) | 2.0 (2.0, 2.9) | 4.5 (4.0, 6.0) | **<0.001** |
| Autoimmune antibody, n (%) | 75(64.1%) | 46 (54.8%) | 29 (87.9%) | **0.001** |
| Sex, Male (n) | 33 | 25 | 8 |  |
| Age (years, x ̅±s) | 45.7±13.0 | 46.1±12.5 | 44.5±15.3 | 0.764 |
| DS(%) | 3 (9.1%) | 1 (4.0%) | 2 (25.0%) | 0.139 |
| Baseline EDSS | 3.5(2.3, 4.5) | 3.0(2.3, 4.3) | 4.8 (2.6, 5.8) | **0.042** |
| Last follow-up EDSS | 2.5 (2.0, 3.8) | 2.0 (2.0, 3.0) | 5.0 (4.1, 6.4) | **<0.001** |
| Autoimmune antibody, n (%) | 20(60.6%) | 13 (52.0%) | 7(87.5%) | 0.108 |

Note:

DS: Diencephalic syndrome; EDSS: Expanded Disability Status Scale.
